# Copper-catalyzed *N*-arylation of amines with aryliodonium ylides in water

**DOI:** 10.3762/bjoc.19.76

**Published:** 2023-07-04

**Authors:** Kasturi U Nabar, Bhalchandra M Bhanage, Sudam G Dawande

**Affiliations:** 1 Department of Chemistry, Institute of Chemical Technology, Nathalal Parekh Marg, Matunga East, Mumbai-400019, Maharashtra, India; 2 Department of Chemistry, Indian Institute of Technology Madras, Chennai-600036, Tamilnadu, India

**Keywords:** amines, arylation, C–N bond formation, iodonium ylide, sustainable

## Abstract

Copper sulfate catalyzed an efficient, inexpensive, and environment-friendly protocol that has been developed for *N*-arylation of amines with 1,3-cyclohexadione-derived aryliodonium ylides in water as a green solvent. Aromatic primary amines substituted with electron-donating as well as electron-withdrawing groups on the aryl ring reacted smoothly with iodonium ylides to give the corresponding diarylamines with good to excellent yields. Also, secondary amines underwent *N*-arylation to deliver tertiary amines with moderate yields.

## Introduction

**Figure 1:**
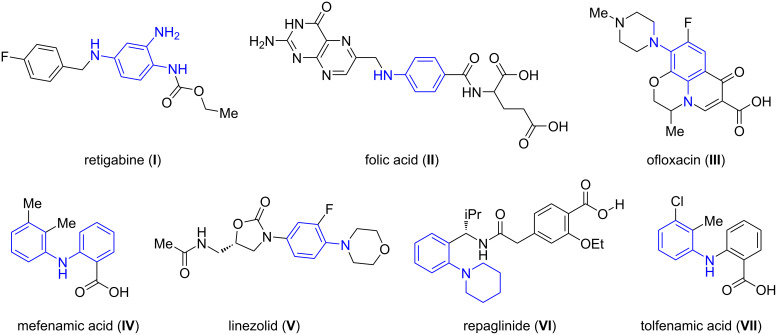
Representative examples of *N*-arylamines.

Arylamines are among the most privileged structural motifs appearing in various natural products, and bioactive molecules [1–2] as well as offer widespread applications in pharmaceuticals, agrochemicals, dyes, and materials science [3–4]. Particularly the pharmaceutical agents containing the arylamine moiety as an integral part of the structural framework includes molecule such as retigabine (**I**) [5], an anticonvulsant used as an adjunctive agent in the treatment of partial seizures, folic acid (**II**) [6], a type of vitamin B12 displaying an important role in metabolism, cell growth and during pregnancy, ofloxacin (**III**) [7], an antibacterial agent, mefenamic acid (**IV**) [8], an anti-inflammatory agent used to treat mild pain, linezolid (**V**) [9], an antibacterial agent, repaglinide (**VI**) [10], used to treat diabetes mellitus type 2, and tolfenamic acid (**VII**) [11], an anti-inflammatory agent (Figure 1).

Owing the importance of arylamines and their biological significance, the surge of development of innovative methods for their synthesis has been always an area of interest [12]. Specifically, the metal-mediated arylation reactions have witnessed significant development since the pioneering work by Ullmann [13] and Goldberg [14]. Despite the significant advances, the main limitations are the harsh reaction conditions such as high temperatures, pressure, and unsustainability which limits their scalability for industrial applications. Further, palladium-catalyzed strategies for C–N bond formation have been extensively explored by various research groups for the *N*-arylation of amines. Specifically, seminal contributions by Buchwald [15] and Hartwig [16] involving the use of palladium complexes as catalysts in the presence of either phosphine or diamine ligands for C–N bond formation. However, these methods suffer from limitations such as moisture sensitivity, the requirement of specific ligands, and the use of expensive palladium catalysts [17]. Also, Chan Lam, Evans, and other research groups have developed copper-catalyzed C–N bond formation reactions by careful tuning of the ligand and base combinations [18–19]. Thereafter, copper-catalyzed C–N bond-formation reactions have experienced unprecedented development due to mild reaction conditions and the low cost of copper salts [20–22].

**Scheme 1:**
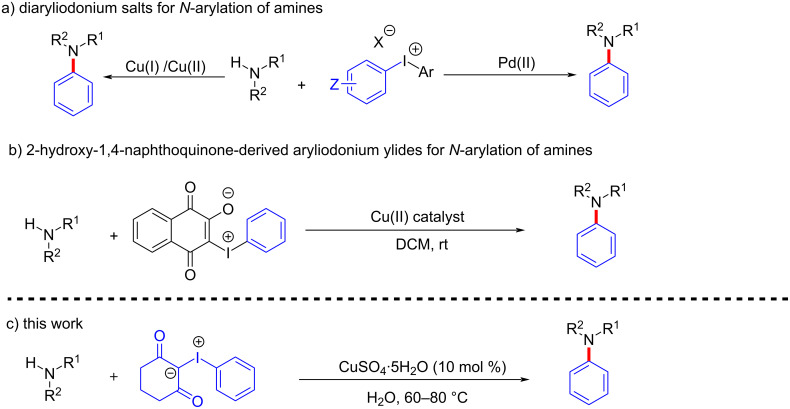
*N*-Arylation of amines with hypervalent iodine reagents.

On the other hand, hypervalent iodine reagents serve as versatile tools in oxidation, C–C, C–X bond formation, rearrangements, and halogenation reactions [23–25]. Due to the nontoxic nature, easier preparation, and handling of the hypervalent iodine reagents, many researchers are attracted to unravel the chemistry and reactivity of these reagents. Amongst different types of hypervalent iodine reagents, diaryliodonium salts are commonly used reagents for the *N*-arylation of nitrogen-containing compounds, particularly for *N*-arylation of amines under catalyst-free conditions either in the presence of additives or at higher temperatures [26–32]. Further, a few reports are also available for the copper and palladium-catalyzed *N*-arylation of primary and secondary aliphatic as well as aromatic amines using diaryliodonium salts as aryl sources [33–35] (Scheme 1a). Similarly, iodonium ylides undergo a wide range of reactions through in situ generation of carbene as a reactive intermediate [36–37]. Also, spirocyclic iodonium ylides have been used for radiolabeling techniques [38]. In 2013, Shibata’s research group reported a novel trifluoromethanesulfonyl iodonium ylide for trifluoromethylthiolation of enamines, indoles, and ketoesters, catalyzed by a copper catalyst [39]. Murphy and co-workers reported blue LED-mediated metal-free cyclopropanation of alkenes with iodonium ylides through a diradical intermediate [40]. However, iodonium ylides are relatively unexplored for the arylation of amines. So far only Spyroudis’s group reported *N*-arylation of amines using iodonium ylides obtained from 2-hydroxy-1,4-naphthoquinone in the presence of a Cu(II) catalyst in dichloromethane as a solvent (Scheme 1b) [41]. Albeit useful, this protocol suffers drawbacks such as low yields, formation of byproducts, limited substrate study, and the use of halogenated solvents. Moreover, the use of economic and eco-friendly solvents such as water have always been an attractive area in organic synthesis.

Therefore, in continuation of our efforts towards the development of sustainable methods for the synthesis of valuable organic molecules, herein, we report a straightforward and efficient method for the *N*-arylation of primary arylamines and secondary amines with 1,3-cyclohexanedione-derived aryliodonium ylides in the presence of copper catalysts in water as a solvent.

## Results and Discussion

To test our hypothesis, we commenced our studies by treatment of aniline (**1a**) with iodonium ylide 2-(phenyl-λ^3^-iodaneylidene)cyclohexane-1,3-dione (**2a**) obtained from 1,3-cyclohexanedione in the presence of copper salts as catalysts. The detailed optimization studies are described in Table 1. Initially, we treated aniline (**1a**, 0.2 mmol) with iodonium ylide **2a** (0.24 mmol) in the presence of 5 mol % of Cu(OTf)_2_ at room temperature in 2 mL of 1,2-dichloroethane (DCE) as a solvent. To our delight, the desired product diphenylamine (**3a**) was formed in 15% yield after 12 hours (Table 1, entry 1). While under similar reaction conditions, with 10 mol % of Cu(OTf)_2_, product **3a** obtained in 35% yield (Table 1, entry 2). When the reaction was carried out at 60 °C the product yield was improved to 49% with complete consumption of starting materials in 4 hours (Table 1, entry 3). Next, to examine the role of the copper catalyst, we screened a variety of Cu(II) salts, namely, Cu(OAc)_2_, CuCl_2_, CuBr_2_, and CuSO_4_·5H_2_O (Table 1, entries 4–7) with maintaining the same reaction conditions. Among these salts, CuSO_4_·5H_2_O afforded a relatively higher yield of the product diphenylamine (**3a**, Table 1, entry 4). Inspired by this observation, we carried out the *N*-arylation of aniline (**1a**) with iodonium ylide **2a** in the presence of 10 mol % of CuSO_4_·5H_2_O as a catalyst in solvents such as tetrahydrofuran, 1,4-dioxane, methanol, water, acetonitrile, *N,N*-dimethylformamide and toluene at 60 °C (Table 1, entries 9–14). The detailed investigation reveals that the arylation of aniline (**1a**) with iodonium ylide **2a** proceeds efficiently in the presence of 10 mol % of CuSO_4_·5H_2_O in water as a solvent at 60 °C to provide diphenylamine (**3a**) with 82% yield (Table 1, entry 11). In addition, we conducted the reaction in the presence of 10 mol % of CuSO_4_·5H_2_O as a catalyst in water as a solvent at room temperature. However, the reaction was sluggish and resulted in only 52% yield after 48 hours (Table 1, entry 15).

**Table 1:** Optimization of reaction conditions^a^.

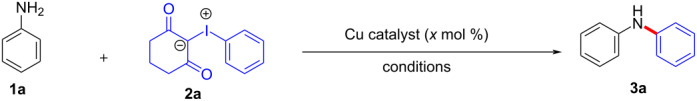					

Entry	Catalyst (mol %)	Solvent	Temp. (°C)	Yield^b^ (%)	Time (h)

1	Cu(OTf)_2_ (5)	DCE	rt	15	12
2	Cu(OTf)_2_ (10)	DCE	rt	35	10
3	Cu(OTf)_2_ (10)	DCE	60	49	4
4	CuSO_4_·5H_2_O (10)	DCE	60	60	2
5	CuCl_2_ (10)	DCE	60	44	2
6	CuBr_2_ (10)	DCE	60	36	2
7	Cu(OAc)_2_ (10)	DCE	60	46	2
8	CuSO_4_·5H_2_O **(**10)	THF	60	25	5
9	CuSO_4_·5H_2_O (10)	1,4-dioxane	60	30	2
10	CuSO_4_·5H_2_O (10)	MeOH	60	54	2
**11**	**CuSO** **_4_** **·5H** **_2_** **O (10)**	**water**	**60**	**82**	**2**
12	CuSO_4_·5H_2_O (10)	MeCN	60	48	4
13	CuSO_4_·5H_2_O (10)	DMF	60	44	2
14	CuSO_4_·5H_2_O (10)	toluene	60	40	2
15	CuSO_4_·5H_2_O (10)	water	rt	52	48

^a^Reaction conditions: aniline (**1a**, 0.2 mmol), ylide **2a** (0.24 mmol), solvent (2 mL), copper catalyst, ^b^isolated yield.

**Scheme 2:**
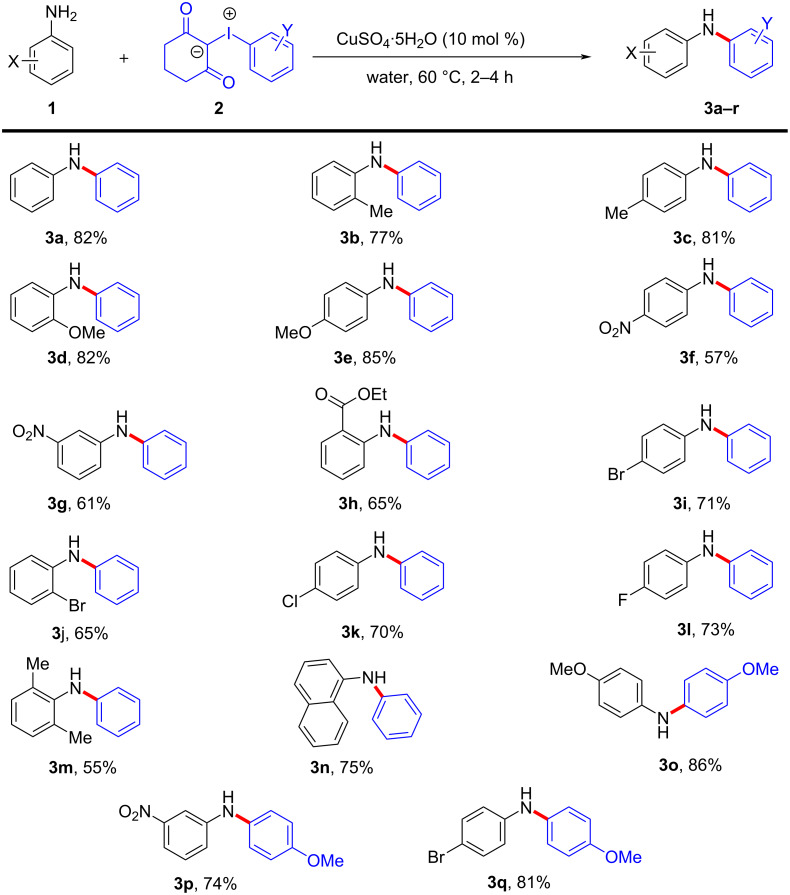
*N*-Arylation of primary amines with iodonium ylide. Reaction conditions: 0.2 mmol aniline **1**, 0.24 mmol iodonium ylide **2,** CuSO_4_·5H_2_O (10 mol %), water (2 mL).

With the optimal reaction conditions in hand, we focused our attention on evaluating the scope and limitations of copper-catalyzed *N*-arylation using iodonium ylide **2** of variably substituted primary arylamines **1**, bearing electron-donating as well as electron-withdrawing groups on the aromatic ring. The results are shown in Scheme 2.

The reaction of *o*-toluidine and *p*-toluidine with ylide **2a** resulted in products **3b** and **3c** with 77% and 81% yields. Next, the electron-rich arylamines *o*-anisidine and *p*-anisidine reacted efficiently with iodonium ylide **2a** under standard conditions to give products **3d** and **3e** with excellent yields. Further, the treatment of arylamines containing electron-withdrawing groups such as *p*-NO_2_, *m*-NO_2,_ and *o*-CO_2_Et with ylide **2a** delivered the corresponding diarylamines **3f–h** in 57–65% yields. Also, the halogen-substituted arylamines such as *p*-bromo, *o*-bromo, *p*-chloro, and *p*-fluoroanilines reacted smoothly to produce the corresponding diarylamine products **3i–l** with 65–73% yields. To identify the effect of steric hindrance we treated 2,6-dimethylaniline under optimal reaction conditions with iodonium ylide **2a**, which reacted sluggishly compared to other arylamines to give the product **3m** in 55% yield. Next, 1-naphthylamine also smoothly underwent *N*-arylation to furnish product **3n** in 75% yield. Then we studied the arylation of primary arylamine with iodonium ylide, 2-((4-methoxyphenyl)-λ^3^-iodaneylidene)cyclohexane-1,3-dione (**2b**) under identical reaction conditions. The arylamines with having *p*-OMe, *m*-NO_2_, and *p*-bromo substituents reacted efficiently with iodonium ylide **2b** to form their diarylamine derivatives **3o–q** with 74–86% yields.

**Scheme 3:**
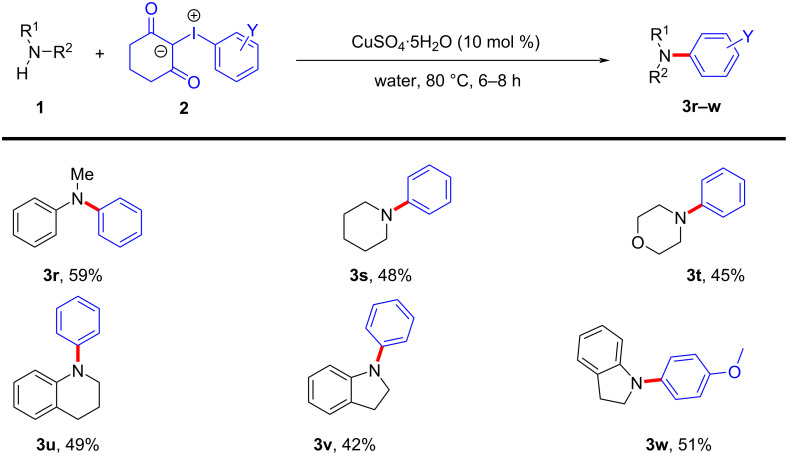
*N*-Arylation of secondary amines with iodonium ylide.

Next, we scrutinized the CuSO_4_·5H_2_O-catalyzed *N*-arylation reaction of secondary amines with iodonium ylide. The secondary amines reacted sluggishly with iodonium ylide **2a** in the presence of 10 mol % of CuSO_4_·5H_2_O catalyst at 60 °C in water giving lower yields. However, with an elevation of the reaction temperature to 80 °C, the secondary amines smoothly proceeded *N*-arylation to give the corresponding tertiary amine derivatives with moderate yields after 6–8 hours. The results are summarised in Scheme 3. The reaction of *N*-methylaniline with iodonium ylide **2a** in the presence of 10 mol % of catalyst delivered *N*-methyl-*N*-phenylaniline (**3r**) as a product with 59% yield. Similarly, the reaction of cyclic secondary amines also resulted in arylation under the optimal reaction conditions to give products **3s–v** with 42–48% yields. The treatment of indoline with iodonium ylide **2b** in the presence of 10 mol % of CuSO_4_·5H_2_O affords product **3w** in 51% yield.

## Conclusion

In summary, we have developed a novel and sustainable method for the C–N bond formation on arylamines with iodonium ylides in the presence of inexpensive copper sulfate as a catalyst in water as a solvent under mild reaction conditions. Diversely substituted primary arylamines, as well as secondary amines, underwent *N*-arylation with aryliodonium ylide obtained from 1,3-cyclohexanedione to furnish moderate to excellent yields of the corresponding secondary and tertiary amines, respectively.

## Supporting Information

File 1 Experimental part.
