# Supplementary material for: Copper-catalyzed N-arylation of amines with aryliodonium ylides in water
Source: Beilstein J Org Chem. 2023 Jul 4;19:1008–14. doi: 10.3762/bjoc.19.76 (PMC10334208; doi:10.3762/bjoc.19.76)
Supplement: File 1 — Experimental part. [file Beilstein_J_Org_Chem-19-1008-s001.pdf]

BEILSTEIN JOURNAL OF ORGANIC CHEMISTRY

Supporting Information

for

Copper-catalyzed *N*-arylation of amines with aryliodonium ylides in water

Kasturi U. Nabar, Bhalchandra M. Bhanage and Sudam G. Dawande

Beilstein J. Org. Chem. **2023,** *19,* 1008–1014. doi:10.3762/bjoc.19.76

Experimental part

License and Terms: This is a supporting information file under the terms of the Creative Commons Attribution License (https://creativecommons.org/licenses/by/4.0). Please note that the reuse, redistribution and reproduction in particular requires that the author(s) and source are credited and that individual graphics may be subject to special legal provisions.
The license is subject to the *Beilstein Journal of Organic Chemistry* terms and conditions: (https://www.beilstein-journals.org/bjoc/terms)

1. General methods	S2
2. Experimental procedures and characterization for iodonium ylide	S3
3. General procedure for N-arylation	S4
4. Characterization data	S4
5. References	S14
6. NMR spectra of products	S14

1. General

All the reactions were performed in clean glassware under nitrogen atmosphere. Solvents were dried using standard methods. 1,2- Dichloroethane and toluene were distilled over calcium hydride. Unless otherwise stated, all the commercial reagents were used as received. Progress of the reaction was monitored by thin-layer chromatography (Merck Silica gel 60 F-254, pre-coated plates on alumina). Column chromatographic purifications were performed on Merck silica gel (100–200 mesh). Melting points were recorded on a digital melting point apparatus and are uncorrected. Spectroscopic characterizations were carried at the Institute of Chemical Technology Mumbai. ^{1}H NMR spectra were recorded on Agilent FT-NMR spectrometers at 400 MHz. ^{13}C NMR spectra were recorded at 101 MHz, and 126 MHz. ^{1}H NMR chemical shifts are reported in ppm relative to the TMS (= 0) and are abbreviated as follows: s (singlet), d (doublet), t (triplet), q (quartet), m (multiplet), br (broad). ^{13}C NMR chemical shifts are reported in ppm relative to the residual $CDCl_3$ signal (= 77.16). IR spectra were recorded on a Shimadzu FT-IR spectrometer.

2. Experimental procedures and characterization for iodonium ylides

2.1 Experimental procedures for the preparation of 2-(phenyl-λ^3-iodaneylidene)-cyclohexane-1,3-dione [2a]:

Obtained by following literature reported method [1]

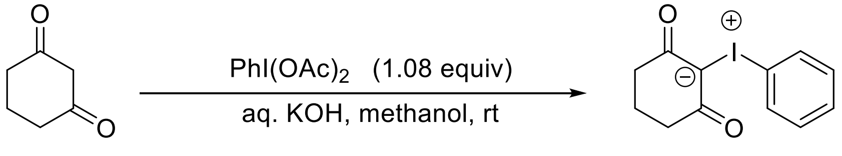

To a solution of cyclic the cyclohex-1,3-dione (14 mmol) in 30 mL methanol, added at room temperature, 20 mL of a 10% aqueous solution of KOH, followed by addition of a solution of diacetoxyiodobenzene (15 mmol) in 40 mL methanol. The reaction mixture was stirred for 2 h at room temperature and then quenched with ice cold water. The resulting white precipitate was filtered and mother liquor was extracted with dichloromethane, then washed with water, dried over anhydrous sodium sulfate, filtered and concentrated in vaccuo. The resultant white solid was mixed with the first crop and the mixture recrystallized from DCM/hexanes.

2.2 Experimental procedures for the preparation of 2-((4-methoxyphenyl)-λ^3-iodaneylidene)cyclohexane-1,3-dione [2b]:

Obtained by following literature reported method [2]

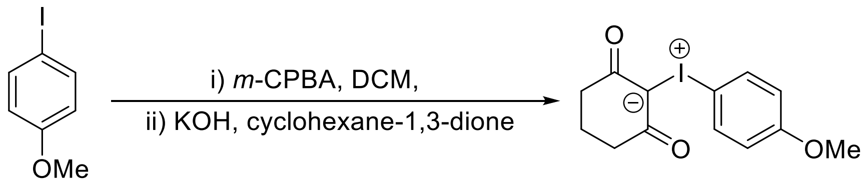

To a solution of 1-iodo-4-methoxybenzene (4.2 mmol, 1g), in dichloromethane, was added *meta*-chloroperbenzoic acid (4.2 mmol, 0.756 g), and the reaction was stirred for 2 hours at ambient temperature. After adding 1.19 g KOH and 1,3 cyclohexane-1,3-dione (4.4 mmol, 0.478 g) the mixture was stirred for another 2 hours at room temperature. The resulting suspension was filtered over celite filter. Then the residue was washed with 30 mL DCM and the filtrates concentrated

under reduced pressure followed by precipitation with hexane. The product was collected by filtration, washed with hexane and dried.

3.A. Typical procedure for preparation of *N*-aryl amines (3a–q)

To a solution of amine **1** (0.2 mmol), iodonium ylide **2** (0.24 mmol) in 2 mL water in a 10 mL round bottom flask equipped with a stir bar 10 mol % $CuSO_4 \cdot 5H_2O$ was added. The reaction mixture was allowed to stir at 60 °C for 2–4 hours (monitored by thin layer chromatography). Then the reaction mixture was diluted with 10 mL ethyl acetate and the organic layer was collected, washed with a saturated aqueous solution of NaCl, and dried over anhydrous Na_2SO_4. Then organic solvents were removed under reduced pressure to obtain a crude residue. The crude residue was purified by silica gel column chromatography to afford corresponding *N*-arylamines (**3a–q**).

3.B. Typical procedure for preparation of *N*-aryl amines (3r–w)

To a solution of amine **1** (0.2 mmol), iodonium ylide **2** (0.24 mmol) in 2 mL water in a 10 mL round bottom flask equipped with a stir bar 10 mol % $CuSO_4 \cdot 5H_2O$ was added. The reaction mixture was allowed to stir at 80 °C for 6–8 hours (monitored by thin layer chromatography). Then the reaction mixture was diluted with 10 mL ethyl acetate and organic layer was collected, washed with saturated aqueous solution of NaCl, and dried over anhydrous Na_2SO_4. Then organic solvents were removed under reduced pressure to obtain a crude residue. The crude residue was purified by silica gel column chromatography to afford corresponding tertiary arylamines (**3r–w**).

4. Characterization data

The obtained spectral data were compared with reported analytical data [3-10]

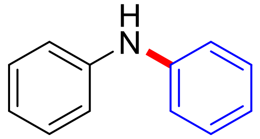

3a

Diphenylamine (3a) [3]: White solid, yield = 82%, m. p. = 54-56°C; **^{1}H NMR (400 MHz, $CDCl_3$)** δ 7.25 (dd, *J* = 8.3, 7.5 Hz, 4H), 7.05 (d, *J* = 7.6 Hz, 4H), 6.92 (t, *J* = 7.3 Hz, 2H), 5.66 (s, 1H); **^{13}C NMR (101 MHz, $CDCl_3$)** δ 143.3, 129.5, 121.1, 118.0; **FTIR (neat):** 3407, 3383, 1592, 1423, 742, 685 cm^{-1}; **LCMS (*m/z*)**: Calculated for $C_{12}H_{12}N$ $[M+H]^+$ = 170.09, observed = 170.11.

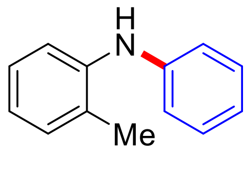

3b

2-Methyl-*N*-phenylaniline (3b) [3]: White solid, yield = 77% m. p. =134-136°C; **^{1}H NMR (400 MHz, $CDCl_3$)** δ 7.35 – 7.15 (m, 4H), 7.15-7.05 (m, 1H), 5.33 (s, 1H), 2.23 (s, 3H); **^{13}C NMR (101 MHz, $CDCl_3$)** δ 144.1, 141.3, 131.0, 129.4, 128.5, 126.9, 122.1, 120.5, 119.0, 117.5, 18.0; **FTIR (neat)**: 3387, 3046, 1582, 1494, 1154, 740, 692 cm^{-1}; **LCMS (*m/z*)**: Calculated for $C_{13}H_{14}N$ $[M+H]^{+}$ = 184.10, observed = 184.23.

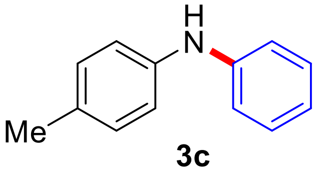

3c

4-Methyl-*N*-phenylaniline (3c) [3]: White solid, yield = 81%, m. p. = 92-94°C; **^{1}H NMR (400 MHz, $CDCl_3$)** δ 7.22 (t, *J* = 8.0 Hz, 2H), 7.07 (d, *J* = 8.0 Hz, 2H), 7.0 (d, *J* = 8.0 Hz, 4H), 6.9 (t, *J* = 6.0 Hz, 1H), 5.0 (s, 1H), 2.3 (s, 3H); **^{13}C NMR (101 MHz, $CDCl_3$)** δ 144.0, 140.4, 131.0, 130.0, 129.4, 120.4, 119.0, 117.0, 20.8; **FTIR (neat)**: 3394, 1594, 1306, 1077, 806, 744, 690 cm^{-1}; **LCMS (*m/z*)**: Calculated for $C_{13}H_{14}N$ $[M+H]^{+}$ = 184.10, observed = 184.23.

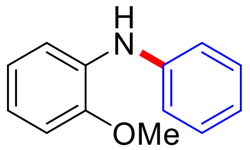

3d

2-Methoxy-*N*-phenylaniline (3d) [5]: Yellow solid, yield = 82%, m. p. = 100-102°C; **^{1}H NMR (400 MHz, $CDCl_3$)** δ 7.37 – 7.26 (m, 3H), 7.16 (d, 2H, *J* = 8Hz), 6.93 (t, *J* = 6.9 Hz, 1H), 6.91 –

6.85 (m, 3H), 6.14 (s, 1H), 3.87 (s, 3H); **^{13}C NMR (101 MHz, CDCl$_3$)** δ 148.4, 142.9, 133.1, 129.4, 121.3, 120.9, 120.0, 118.7, 114.8, 110.7, 55.7; **FTIR (neat)**: 3407, 3047, 1453, 1295, 1025, 746, 692 cm^{-1}; **LCMS (*m/z*)**: Calculated for $C_{13}H_{14}NO$ $[M+H]^+$ = 200.10, observed 200.12.

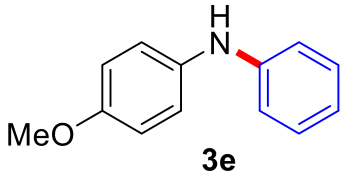

3e

4-Methoxy-*N*-phenylaniline (3e) [3]: White solid, yield = 85%, m. p. = 108-110°C; **^{1}H NMR (400 MHz, CDCl$_3$)** δ 7.12 (t, *J* = 7.5 Hz, 2H), 6.97 (d, *J* = 8.5 Hz, 2H), 6.84 – 6.71 (m, 5H), 5.39 (s, 1H), 3.70 (s, 3H); **^{13}C NMR (101 MHz, CDCl$_3$)** δ 155.4, 145.3, 135.9, 129.4, 122.3, 119.7, 115.8, 114.8, 55.7; **FTIR (neat):** 3386, 2958, 2924, 1499, 1235, 1151, 1032, 742, 694 cm^{-1}; **LCMS (*m/z*)**: Calculated for $C_{13}H_{14}NO$ $[M+H]^+$ = 200.10, observed 200.12.

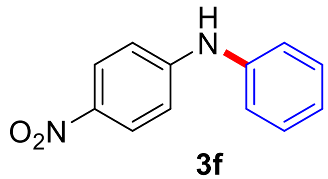

3f

4-Nitro-*N*-phenylaniline (3f) [8]: Yellow solid, yield = 57%, m. p. = 134-136°C; **^{1}H NMR (400 MHz, CDCl$_3$)** δ 8.05 (d, *J* = 8.0 Hz, 2H), 7.32 (t, *J* = 7.9 Hz, 2H), 7.26 – 7.13 (m, 3H), 6.87 (d, *J* = 8.8 Hz, 2H), 6.22 (s, 1H); **^{13}C NMR (101 MHz, CDCl$_3$)** δ 150.3, 139.7, 129.9, 126.4, 124.8, 122.1, 121.8, 113.9; **FTIR (neat)**: 3341.62, 1582, 1275, 1091, 877, 748 cm^{-1}; **LCMS (*m/z*)**: Calculated for $C_{12}H_{11}N_2O_2$ $[M+H]^+$ = 215.07, observed 215.20.

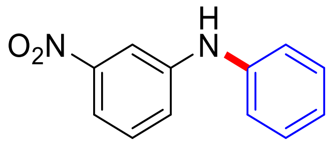

3g

3-Nitro-*N*-phenyl aniline (3g) [8]: White solid, yield= 61%, m. p. = 84-86 °C; **^{1}H NMR (400 MHz, $CDCl_3$)** δ 7.76 (s, 1H), 7.61 (d, J = 7.4 Hz, 1H), 7.41-7.15 (m , 4H), 7.09 – 6.96 (m, 3H), 5.89 (s, 1H); **^{13}C NMR (101 MHz, , $CDCl_3$)** δ 149.5, 145.2, 141.1, 130.1, 129.8, 123.3, 122.0, 120.0, 114.8, 110.4; **FTIR (neat)**: 3391, 3012, 1496, 1260, 1028, 748, 692 cm^{-1}; **LCMS (*m/z*)**: Calculated for $C_{12}H_{11}N_2O_2$ $[M+H]^+$ = 215.07, observed 215.20.

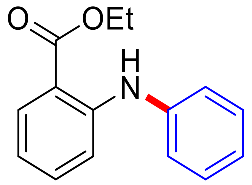

3h

Ethyl 2-(phenylamino)benzoate (3h) [9]: Viscous liquid, yield = 65%; **^{1}H NMR (400 MHz, $CDCl_3$)** δ 9.50 (s, 1H), 7.98 (d, *J* = 8.0 Hz, 1H), 7.42-7.15 (m, 6H), 7.07 (t, *J* = 8.0 Hz, 1H), 6.72 (t, *J* = 8.0 Hz 1H), 4.35 (q, *J* = 8.0 Hz, 2H), 1.40 (t, *J* =6.0 Hz, 3H); **^{13}C NMR (101 MHz, $CDCl_3$)** δ 168.6, 148.1, 141.0, 134.1, 131.8, 129.5, 123.6, 122.6, 117.2, 114.2, 112.4, 60.8, 14.5; **FTIR (neat):** 3360, 2988, 1714, 1600, 1510, 1176, 842, 730 cm^{-1}; **LCMS (*m/z*)**: Calculated for $C_{15}H_{16}NO_2$ $[M+H]^+$ = 242.12, observed 242.43.

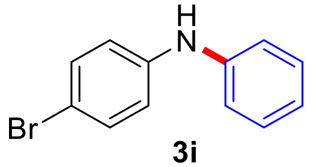

3i

4-Bromo-*N*-phenylaniline (3i) [3]: White solid, yield = 71%, m. p. = 86-88 °C; **^{1}H NMR (400**

MHz, CDCl3) δ 7.34 (d, *J* = 8.0 Hz, 2H), 7.27 (t, *J* = 8.0 Hz, 2H), 7.05 (d, *J* = 8.0 Hz, 2H), 7.02-6.87 (m, 3H), 5.66 (s, 1H); **^{13}C NMR (101 MHz, CDCl3)** δ 142.6, 142.58, 132.3, 129.6, 121.8, 119.2, 118.5, 112.8; **FTIR (neat):** 3400, 2923, 2853, 1578, 1480, 1310, 1070, 747, 689 cm^{-1}; **LCMS (*m/z*)**: Calculated for $C_{12}H_{11}BrN$ $[M+H]^+$ = 248.00, observed 248.16

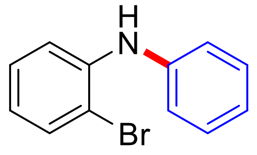

3j

2-Bromo-*N*-phenyl aniline (3j) [3]: White solid, yield = 65%, m. p. = 52-54°C; **^{1}H NMR (400 MHz, CDCl3)** δ 7.42 (dd, *J* = 7.9, 1.3 Hz, 1H), 7.21 (dd, *J* = 11.2, 4.5 Hz, 2H), 7.15 (dd, *J* = 8.1, 1.3 Hz, 1H), 7.09 – 6.87 (m, 3H), 6.94 (t, *J* = 7.3 Hz, 1H), 6.67 – 6.59 (m, 1H), 5.99 (s, 1H); **^{13}C NMR (101 MHz, CDCl3)** δ 141.7, 137.6, 133.1, 129.6, 128.2, 122.8, 121.0, 120.4, 115.9, 112.3; **FTIR (neat):** 3402, 3050, 2926, 1510, 1264, 1021, 732, 702, 694 cm^{-1}; **LCMS (*m/z*)**: Calculated for $C_{12}H_{11}BrN$ $[M+H]^+$ = 248.00, observed 248.16.

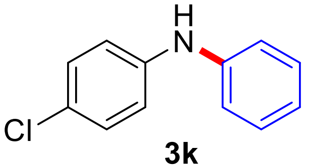

3k

4-Chloro-*N*-phenylaniline (3k) [3]: White solid, yield = 70%, m. p. = 66-68 °C **^{1}H NMR (400 MHz, CDCl3)** δ 7.33 – 7.19 (m, 4H), 7.05-6.95 (m, 5H), 5.66 (s, 1H); **^{13}C NMR (101 MHz, CDCl3)** δ 142.8, 142.0, 129.6, 129.4, 125.6, 121.6, 118.9, 118.2; **FTIR (neat):** 3401, 2922, 1495, 1305, 1088, 748, 690 cm^{-1}; **LCMS (*m/z*)**: Calculated for $C_{12}H_{11}ClN$ $[M+H]^+$ = 204.05, observed 204.16.

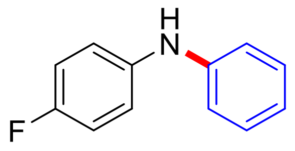

3l

4-Flouro-*N*-phenyl aniline (**3l) [3]:** White solid, yield = 73%, m. p. = 36-68 °C; **^{1}H NMR (400 MHz, CDCl$_3$**) δ 7.78 – 7.61 (m, 1H), 7.28 – 7.23 (m, 2H), 7.11 – 6.94 (m, 5H), 6.94 – 6.87 (m, 1H), 5.58 (s, 1H); **^{13}C NMR (101 MHz, CDCl$_3$)** δ 158.2 (d), 157.0, 144.1, 138.9 (d), 129.5, 120.7 (d), 116.77, 115.77 (d); **FTIR (neat)**: 3420, 2988, 1597, 1508, 1264, 764, 733 cm^{-1}; **LCMS (*m/z*)**: Calculated for $C_{12}H_{11}FN$ $[M+H]^+$ = 187.08, observed 188.14.

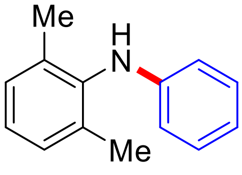

3m

2,6-Dimethyl-*N*-phenylaniline (3m) [3]: White solid, yield = 55%, m. p. = 56-58°C; **^{1}H NMR (400 MHz, CDCl$_3$)** δ 7.20 – 6.89 (m, 5H), 6.65 (s, 1H), 6.41 (d, *J* = 6.5 Hz, 2H), 5.08 (s, 1H), 2.12 (s, 6H); **^{13}C NMR (101 MHz, CDCl$_3$)** δ 146.4, 138.4, 136.0, 129.4, 128.7, 125.9, 118.3, 113.7, 18.34; **FTIR (neat)**: 3391, 3044, 1589, 1495, 1193, 743, 691 cm^{-1}; **LCMS (*m/z*)**: Calculated for $C_{14}H_{16}N$ $[M+H]^+$ = 198.12, observed 198.14.

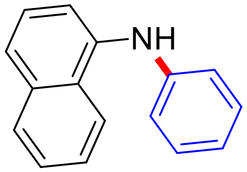

3n

***N*-Phenylnaphthalen-1-amine** (**3n) [3]:** Brown solid, yield = 75%, m. p. = 62-64°C; **^{1}H NMR (400 MHz, CDCl$_3$)** δ 8.00 (d, *J* = 7.6 Hz, 1H), 7.85 (d, *J* = 7.3 Hz, 1H), 7.55 (d, *J* = 7.0 Hz, 1H),

7.50 – 7.42 (m, 2H), 7.42 – 7.32 (m, 2H), 7.24 (t, J = 7.2 Hz, 2H), 6.97 (d, J = 7.6 Hz, 2H), 6.93 – 6.85 (m, 1H), 5.91 (s, 1H); **^{13}C NMR (101 MHz, CDCl$_3$)** δ 144.9, 138.9, 134.8, 129.5, 128.7, 127.9, 126.2, 126.1, 125.8, 123.1, 122.0, 120.6, 117.5, 116.0; **FTIR (neat)**: 3395, 3050, 1591, 1263, 748, 692 cm^{-1}; **LCMS (*m/z*)**: Calculated for $C_{16}H_{14}N$ $[M+H]^+$ = 220.10, observed 220.21.

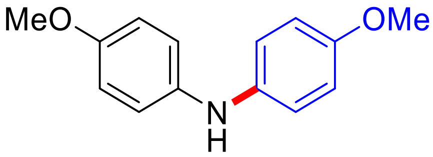

3o

Bis(4-methoxyphenyl)amine (3o) [3]: White solid, yield = 86%, m. p. = 100-102°C; **^{1}H NMR (400 MHz, CDCl$_3$)** δ 7.55 (d, J = 8.5 Hz, 1H), 7.06 – 6.76 (m, 7H), 6.67 (d, J = 8.5 Hz, 1H), 3.77 (s, 6H); **^{13}C NMR (101 MHz, CDCl$_3$)** δ 138.3, 119.8, 116.5, 114.9, 55.8; **FTIR (neat)**: 3422, 2920, 1509, 1240, 1178, 1030, 762, 750 cm^{-1}; **LCMS (*m/z*)**: Calculated for $C_{14}H_{16}NO_2$ $[M+H]^+$ = 230.11, observed 230.24.

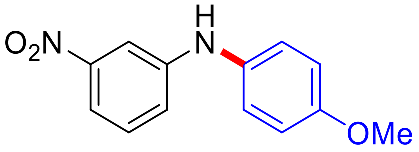

3p

***N*-(4-Methoxyphenyl)-3-nitroaniline (3p) [8] :** Yellow solid, yield= 74%, m. p. = 116-118 °C; **^{1}H NMR (400 MHz, CDCl$_3$)** δ 7.64 (s, 1H), 7.59 (d, J = 7.9 Hz, 1H), 7.35-7.22 (m, 1H), 7.15-7.03 (m, 3H), 6.92 (d, J = 8.7 Hz, 2H), 5.77 (s, 1H), 3.82 (s, 3H); **^{13}C NMR (101 MHz, CDCl$_3$)** δ 156.8, 149.6, 147.2, 133.6, 130.0, 124.4, 120.4, 115.1, 113.6, 108.7, 55.7; **FTIR (neat)**: 3380, 2926, 1511, 1338, 1033, 834, 734, 675 cm^{-1}; **LCMS (*m/z*)**: Calculated for $C_{13}H_{13}N_2O_3$ $[M+H]^+$ = 245.08, observed 245.29.

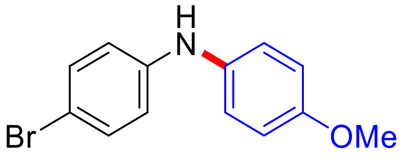

3q

4-Bromo-*N*-(4-methoxyphenyl)aniline (3q) [5]: White solid, yield = 81%, m. p. = 88-90 °C; **^{1}H NMR (400 MHz, CDCl$_3$)** δ 7.27 (d, *J* = 8.8 Hz, 2H), 7.04 (d, *J* = 8.7 Hz, 2H), 6.86 (d, *J* = 8.9 Hz, 2H), 6.75 (d, *J* = 8.8 Hz, 2H), 5.48 (s, 1H), 3.79 (s, 3H); **^{13}C NMR (101 MHz, CDCl$_3$)** δ 155.8, 144.6, 138.3, 132.2, 122.9, 117.1, 116.5, 114.9, 55.7; **FTIR (neat)**: 3418, 2958, 1506, 1296, 1174, 812, 750, 696 cm^{-1}; **LCMS (*m/z*)**: Calculated for $C_{13}H_{13}BrNO$ $[M+H]^+$ = 278.01, observed 278.12.

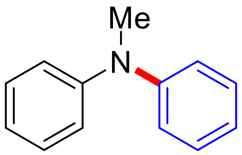

3r

***N*-Phenyl-*N*-methylaniline (3r) [10]:** Viscous liquid, yield = 59%; **^{1}H NMR (400 MHz, CDCl$_3$)** δ 7.19 (t, *J* = 7.5 Hz, 4H), 6.94 (d, *J* = 7.7 Hz, 4H), 6.87 (t, *J* = 6.8 Hz, 2H), 3.23 (s, 3H); **^{13}C NMR (101 MHz, CDCl$_3$)** δ 149.2, 129.3, 121.4, 120.6, 40.4; **FTIR (neat)**: 3036, 2923, 1590, 1494, 1341, 1252, 742, 691 cm^{-1}; **LCMS (*m/z*)**: Calculated for $C_{13}H_{14}N$ $[M+H]^+$ = 184.10, observed $[M+H]^+$ = 184.11.

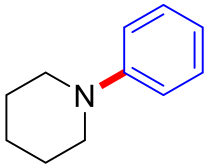

3s

***N*-Phenylpiperidine** (**3s) [6]:** Viscous liquid, yield= 48%; **^{1}H NMR (400 MHz, CDCl$_3$)** δ 7.16 (d, *J* = 7.0 Hz, 2H), 6.87 (d, *J* = 7.1 Hz, 2H), 6.78 – 6.70 (m, 1H), 3.09 (t, *J* = 10.4 Hz, 4H), 1.56 – 1.45 (m, 4H), 0.86 – 0.72 (m, 2H); **^{13}C NMR (101 MHz, CDCl$_3$)** δ 152.5, 129.1, 119.4, 116.7, 50.9, 26.0, 24.5; **FTIR (neat)**: 2921, 2852, 1462, 1259, 1017, 796, 721 cm^{-1}; **LCMS (*m/z*)**: Calculated for $C_{11}H_{16}N$ $[M+H]^+$ = 162.13, observed 162.10.

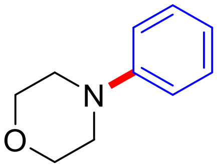

3t

***N*-Phenylmorpholine** (**3t) [3]:** Viscous liquid, yield = 45%; **^{1}H NMR (400 MHz, CDCl$_3$)** δ 7.26 – 7.15 (m, 2H), 6.88 – 6.75 (m, 3H), 3.84 – 3.74 (m, 4H), 3.15 – 3.04 (m, 4H); **^{13}C NMR (101 MHz, CDCl$_3$)** δ 151.4, 129.3, 120.2, 115.8, 67.1, 49.6; **FTIR (neat)**: 2988, 1264, 1035, 764, 732 cm^{-1}; **LCMS (*m/z*)**: Calculated for $C_{10}H_{14}NO$ $[M+H]^+$ = 164.10, observed = 164.10.

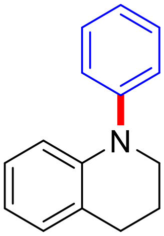

3u

***N*-Phenyl-1,2,3,4-tetrahydroquinoline (3u) [4]:** Viscous liquid, yield = 49%; **^{1}H NMR (400 MHz, CDCl$_3$)** δ 7.26 (t, *J* = 7.8 Hz, 2H), 7.15 (d, *J* = 8.4 Hz, 2H), 7.00 (t, *J* = 7.4 Hz, 1H), 6.96

(d, J = 7.3 Hz, 1H), 6.84 (t, J = 7.7 Hz, 1H), 6.66 (d, J = 8.3 Hz, 1H), 6.62 (t, J = 7.3 Hz, 1H), 3.57 – 3.52 (m, 2H), 2.77 (t, J = 6.3 Hz, 2H), 1.96 (dt, J = 12.3, 6.2 Hz, 2H); **^{13}C NMR (101 MHz, $CDCl_3$)** δ 148.5, 144.6, 129.5, 126.5, 124.8, 124.7, 123.7, 118.4, 115.9, 114.2, 51.0, 27.9, 22.9; **FTIR (neat)**: 2928, 1588, 1470, 1360, 748 cm^{-1}; **LCMS (*m/z*)**: Calculated for $C_{15}H_{16}N$ $[M+H]^+$ = 210.35, observed 210.12.

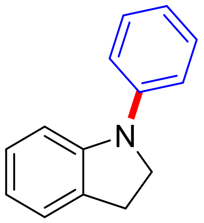

3v

***N*-Phenylindoline** (**3v**) **[4]:** Yellow solid, yield = 42%, m. p. = 52-54 °C; **^{1}H NMR (400 MHz, $CDCl_3$)** δ 7.67 (d, J = 7.7 Hz, 1H), 7.55-7.25 (m, 2H), 7.25 – 7.11 (m, 3H), 7.10-7.00 (m, 1H), 6.94 (t, J = 7.2 Hz, 1H), 6.73 (t, J = 7.3 Hz, 1H), 3.90 (t, J = 8.4 Hz, 2H), 3.08 (t, J = 8.3 Hz, 2H); **^{13}C NMR (101 MHz, $CDCl_3$)** δ 147.1, 137.6, 130.3, 129.2, 127.1, 125.1, 121.0, 118.9, 117.7, 108.2, 52.2, 28.3; **FTIR (neat):** 2923, 1593, 1496, 1364, 748, 692 cm^{-1}; **LCMS (*m/z*)**: Calculated for $C_{14}H_{14}N$ $[M+H]^+$ = 196.10, observed 196.25.

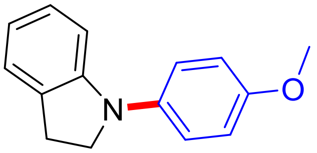

3w

1-(4-Methoxyphenyl)indoline (3w) [7]: Yellow solid, yield= 51%, m. p. = 128-130°C; **^{1}H NMR (400 MHz, $CDCl_3$)** δ 7.20 – 7.10 (m, 3H), 7.02 (t, J = 7.4 Hz, 1H), 6.93 – 6.86 (m, 3H), 6.69 (t, J = 7.2 Hz, 1H), 3.85 (t, J = 8.4 Hz, 2H), 3.79 (s, 3H), 3.09 (t, J = 8.3 Hz, 2H); **^{13}C NMR (101 MHz, $CDCl_3$)** δ 154.9, 148.7, 138.1, 130.8, 127.2, 125.0, 120.8, 118.3, 114.7, 107.5, 55.7, 53.2, 28.4;

FTIR (neat): 3047, 2850, 1508, 1240, 1034, 824, 740 cm^{-1}; **LCMS (*m/z*)**: Calculated for $C_{15}H_{16}NO$ $[M+H]^+$ = 226.12, observed 226.11.

5. References:

1. Moriarty, R. M.; Tyagi, S.; Ivanov, D.; Constantinescu, M. *J. Am. Chem. Soc.* **2008**, *130*, 7564.
2. Cardinale, J.; Ermert, J. *Tetrahedron Lett.* **2013**, *54*, 2067.
3. Zhu, X.; Zhang, Q.; Su, W. *RSC Adv.* **2014**, *4*, 22775.
4. Joe, C. L.; Doyle, A. G. *Angew. Chem. Int. Ed.* **2016**, *55*, 4040.
5. Han, Y.; Zhang, M.; Zhang, Y.; Zhang, Z. H *Green Chem.* **2018**, *20*, 4891.
6. He, H.; Zhang, Z.; Tang, H.-B.; Xu, Y.; Xu, X.; Cao, Z.-Y.; Xu, H.; Li, Y. *Org. Chem. Front.*, **2022**, *9,* 4875.
7. Zihao, L.; Meng, F.; Zhang, J.; Xie, J.-W.; Dai, **B** *Org. Biomol. Chem.*, **2016**, *14*, 10861.
8. Guo, X.; Rao, H.; Fu, H.; Jiang, Y.; Zhao, Y. *Adv. Syn. & Catal.* **2006**, *348*, 2197.
9. Barros, M. T.; Dey, S. S.; Maycock, C. D.; Rodrigues, P. *Chem. Commun.*, **2012**, *48*, 10901.
10. Bracher, F.; Popp, T. *Synthesis* **2015**, *47*, 3333.

6. ^{1}H and ^{13}C NMR Spectra

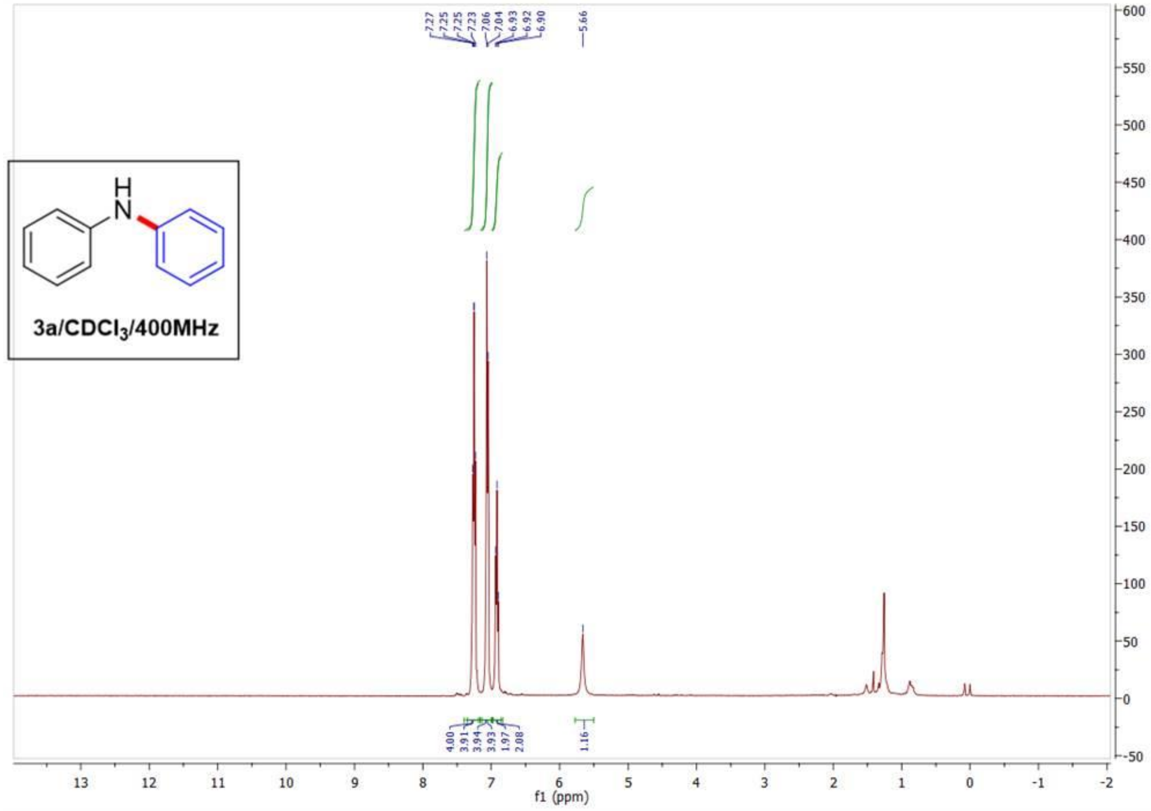

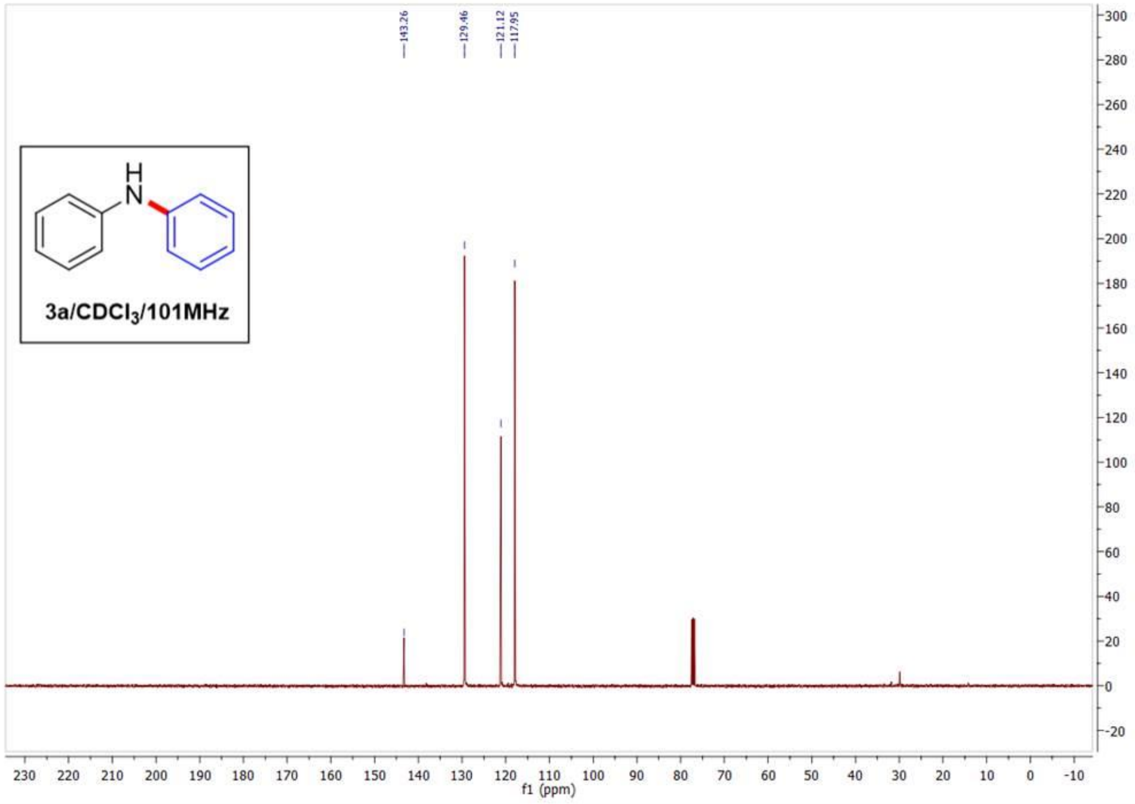

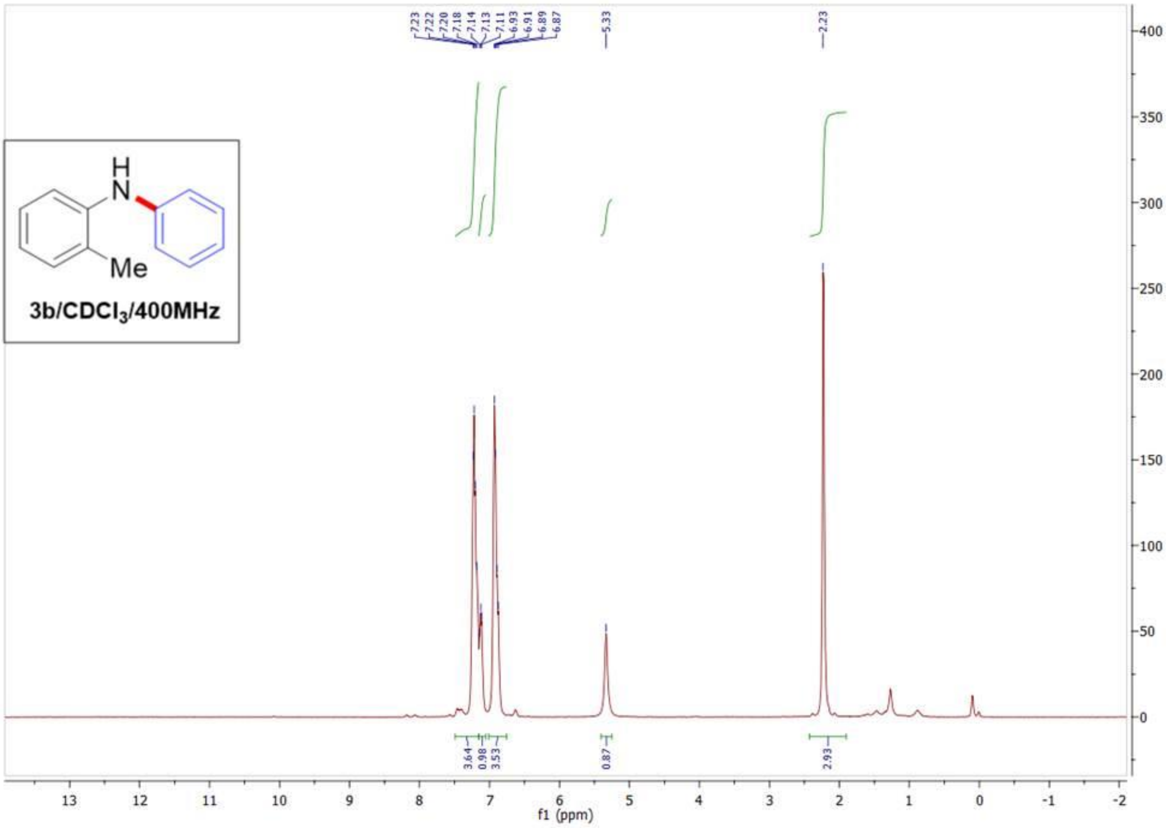
3b/CDCl3/400MHz
Me
H
N
f1 (ppm)

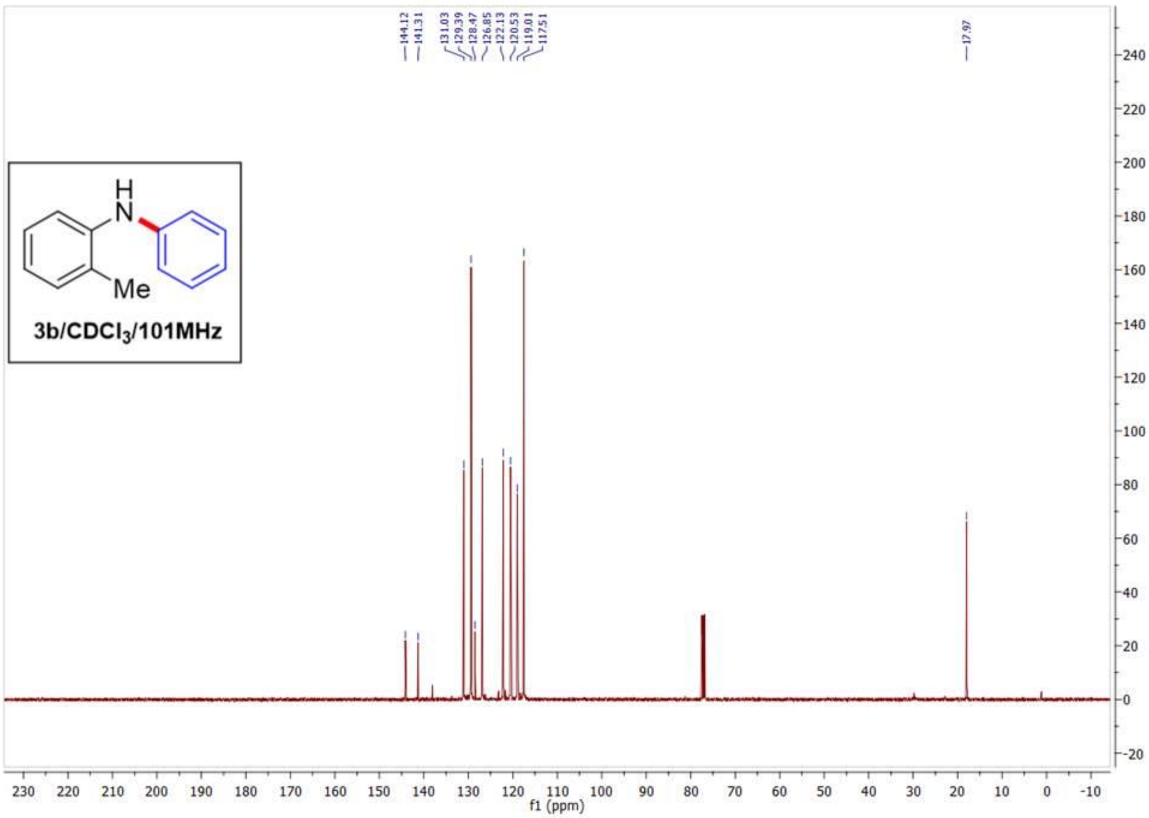
3b/CDCl3/101MHz
Me
H
N
144.12
141.31
131.03
129.39
128.47
126.85
122.13
120.53
119.01
117.51
17.97
f1 (ppm)

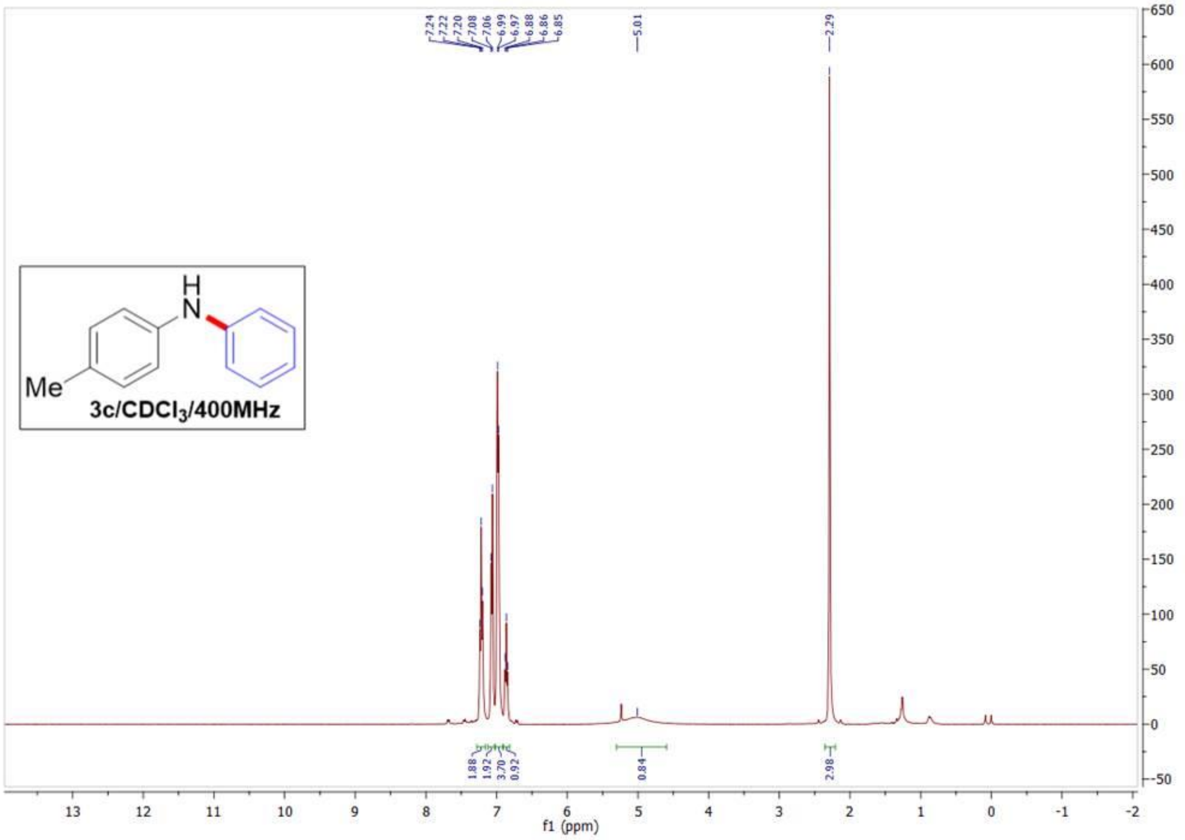
7.24
7.22
7.20
7.08
7.06
6.99
6.97
6.88
6.86
6.85
5.01
2.29
H
N
Me
3c/CDCl$_3$/400MHz
1.88
1.92
3.70
0.92
0.84
2.98
f1 (ppm)

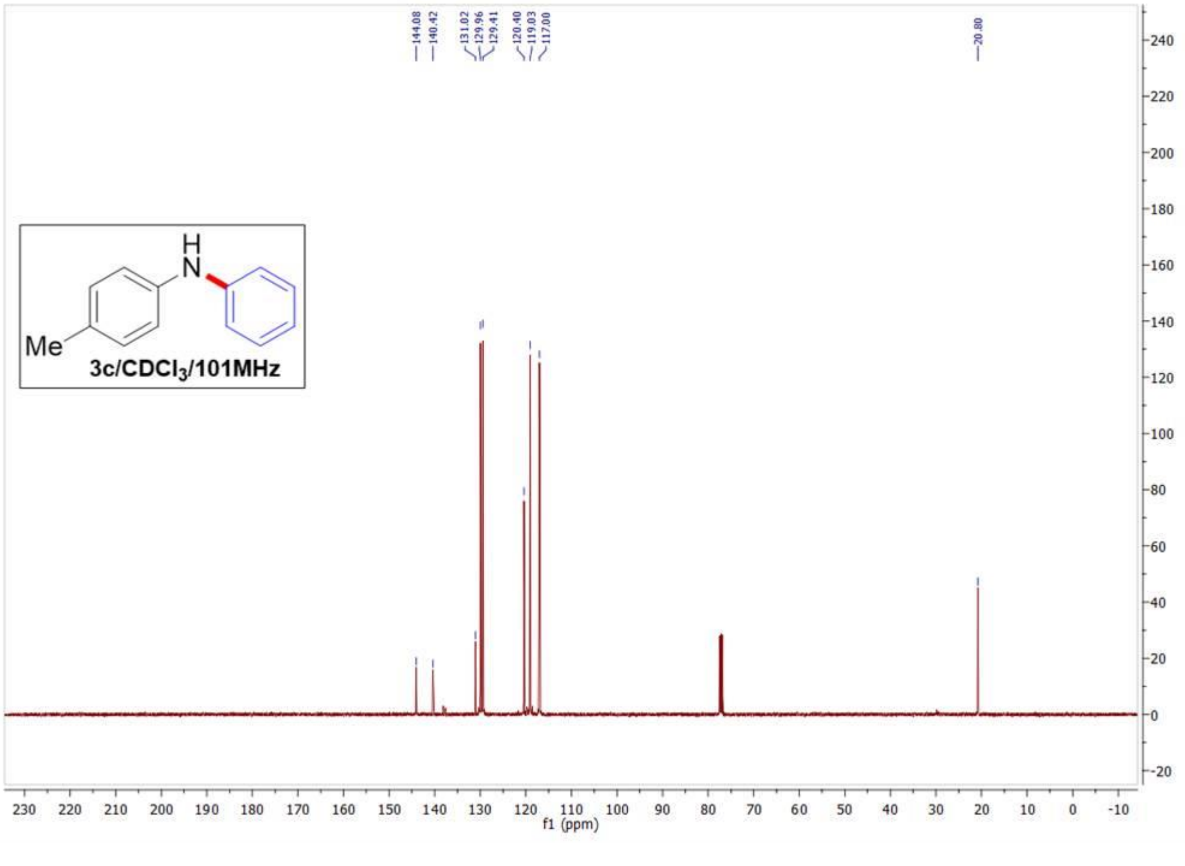
144.08
140.42
131.02
129.96
129.41
120.40
119.03
117.00
20.80
H
N
Me
3c/CDCl$_3$/101MHz
f1 (ppm)

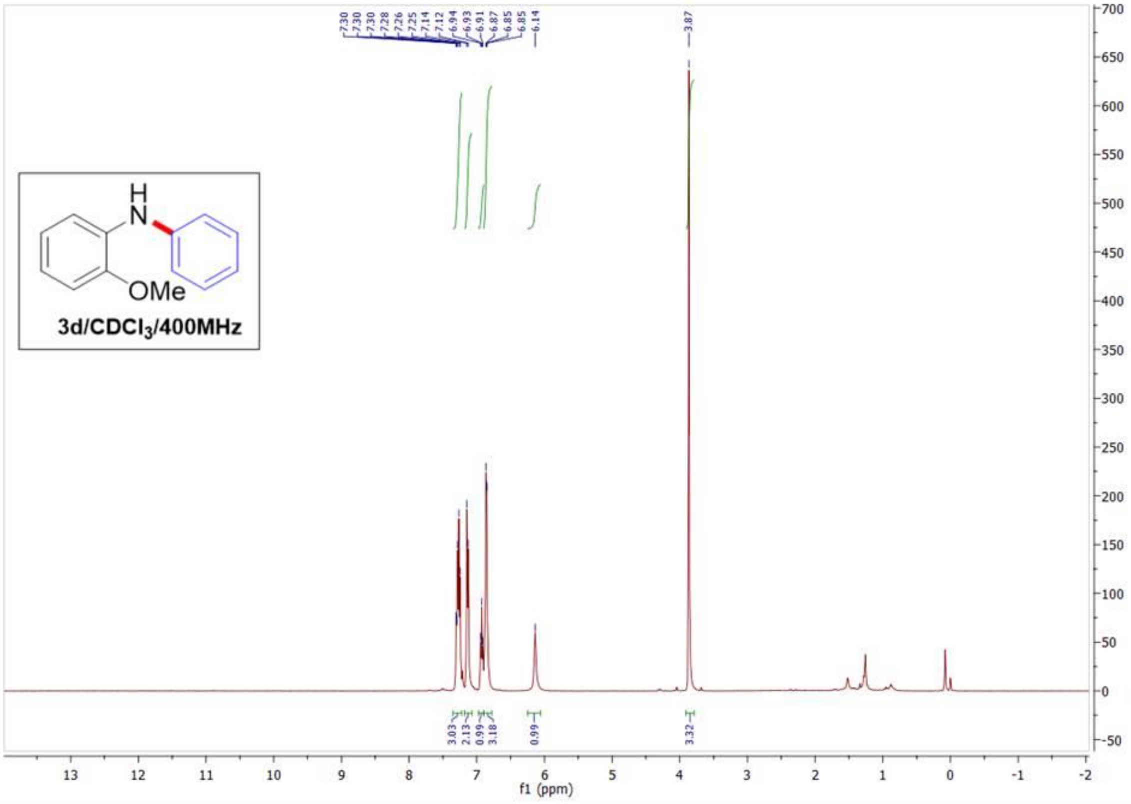
3d/CDCl3/400MHz
f1 (ppm)

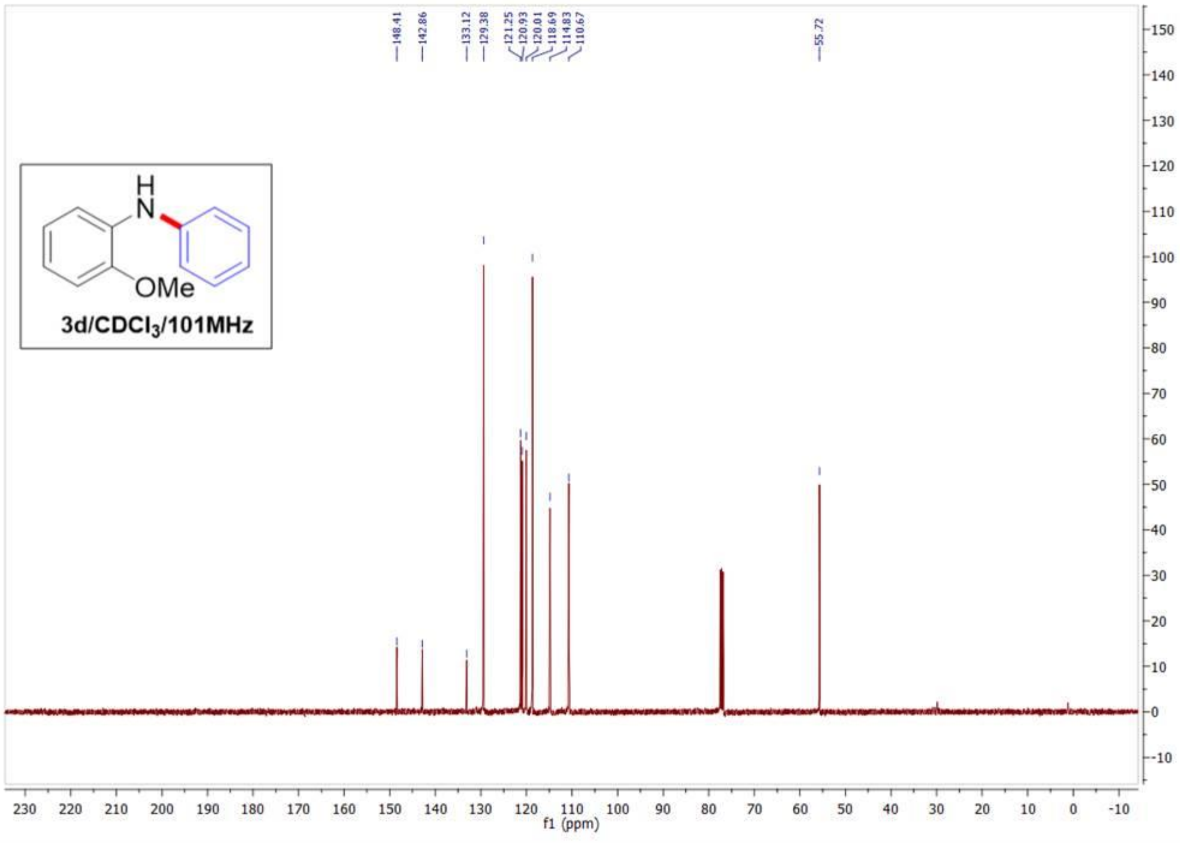
3d/CDCl3/101MHz
f1 (ppm)

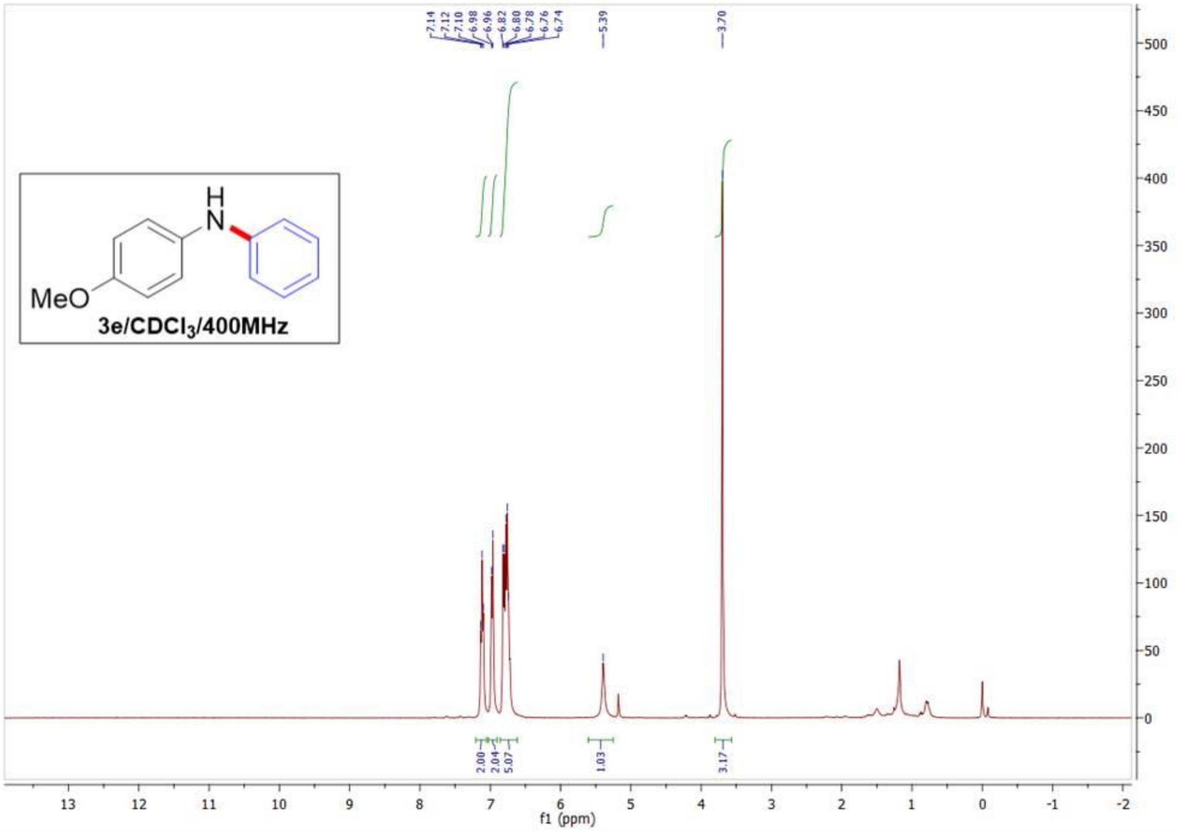

7.14
7.12
7.10
6.98
6.96
6.82
6.80
6.78
6.76
6.74
5.39
3.70
MeO
H
N
3e/CDCl3/400MHz
2.00
2.04
5.07
1.03
3.17
f1 (ppm)

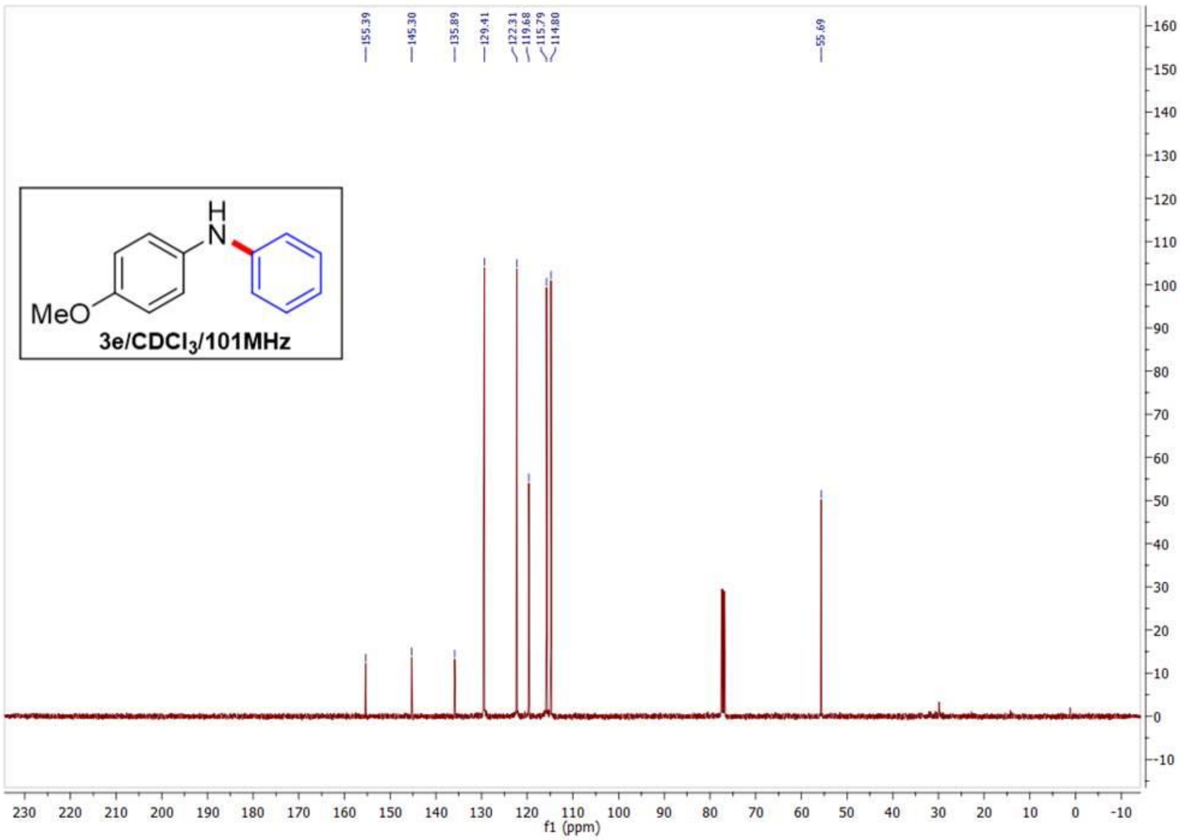

155.39
145.30
135.89
129.41
122.31
119.68
115.79
114.80
55.69
MeO
H
N
3e/CDCl3/101MHz
f1 (ppm)

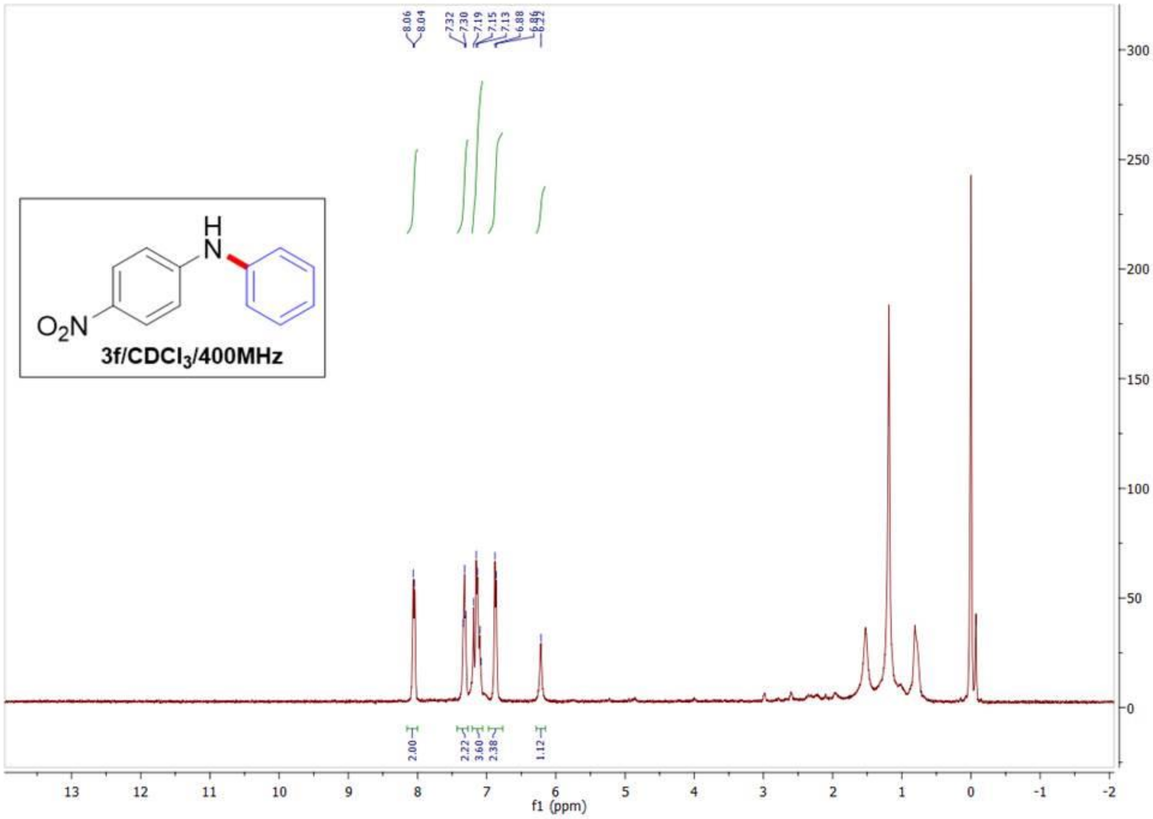
8.06
8.04
7.32
7.30
7.19
7.15
7.13
6.88
6.86
6.22
H
N
O2N
3f/CDCl3/400MHz
2.00
2.22
3.60
2.38
1.12
f1 (ppm)

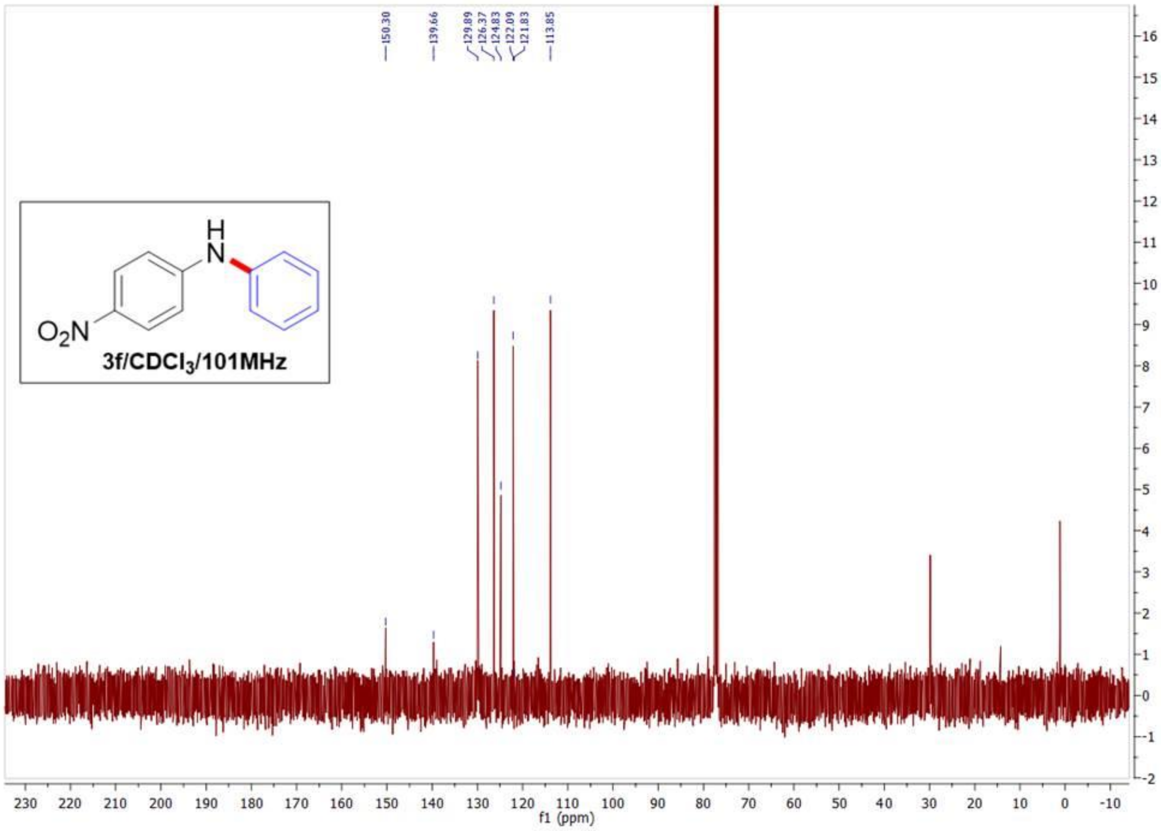
150.30
139.66
129.89
126.37
124.83
122.09
121.83
113.85
H
N
O2N
3f/CDCl3/101MHz
f1 (ppm)

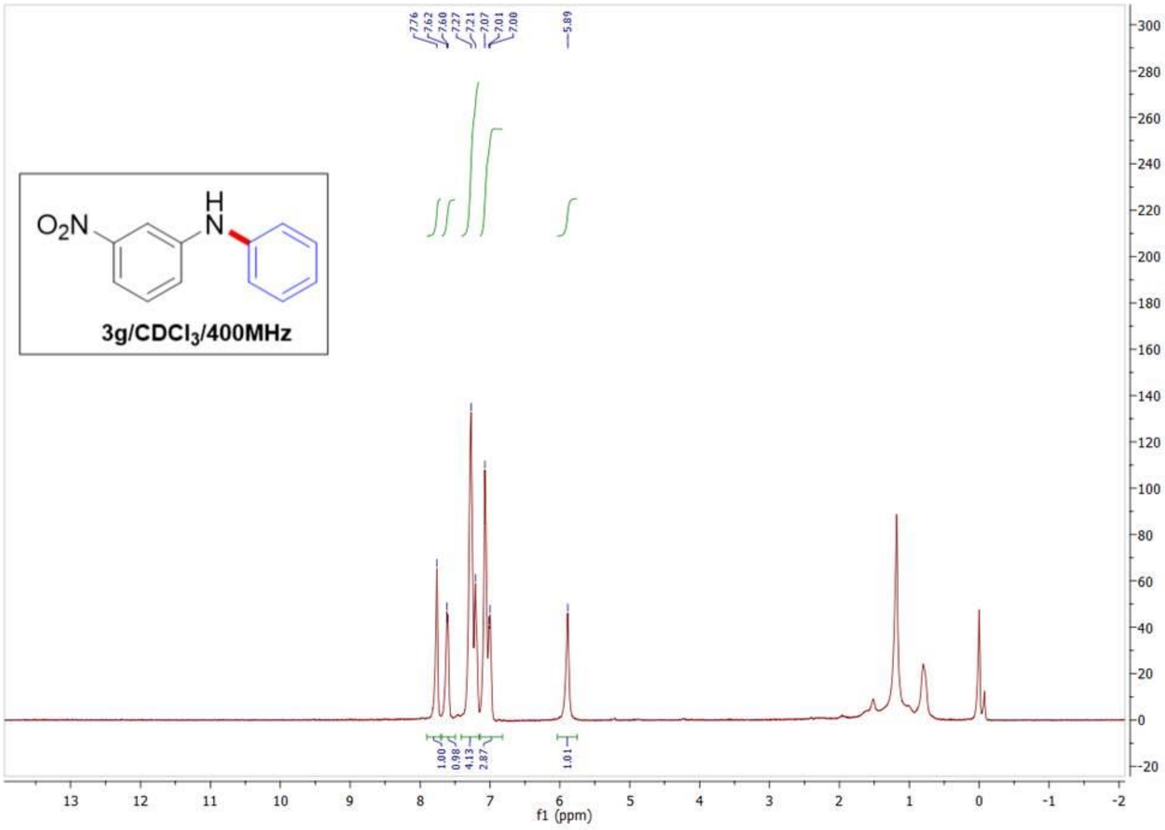
3g/CDCl3/400MHz
f1 (ppm)

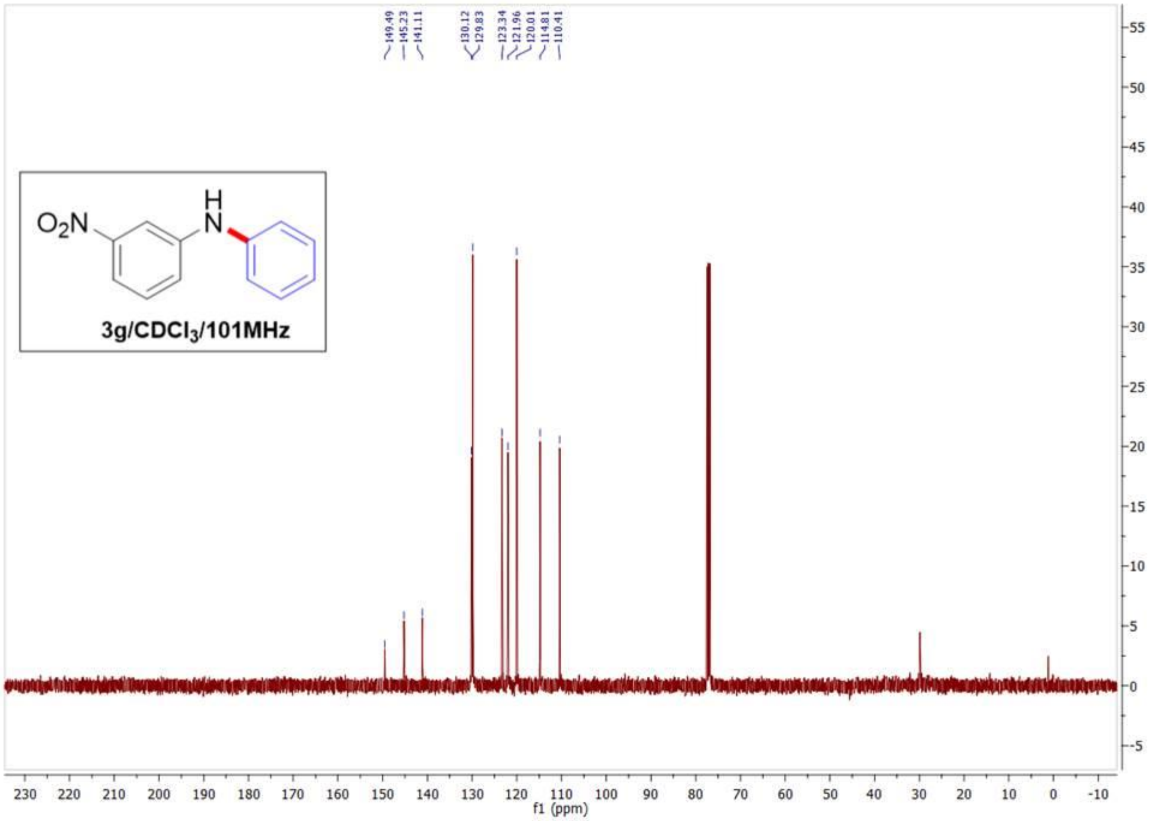
3g/CDCl3/101MHz
f1 (ppm)

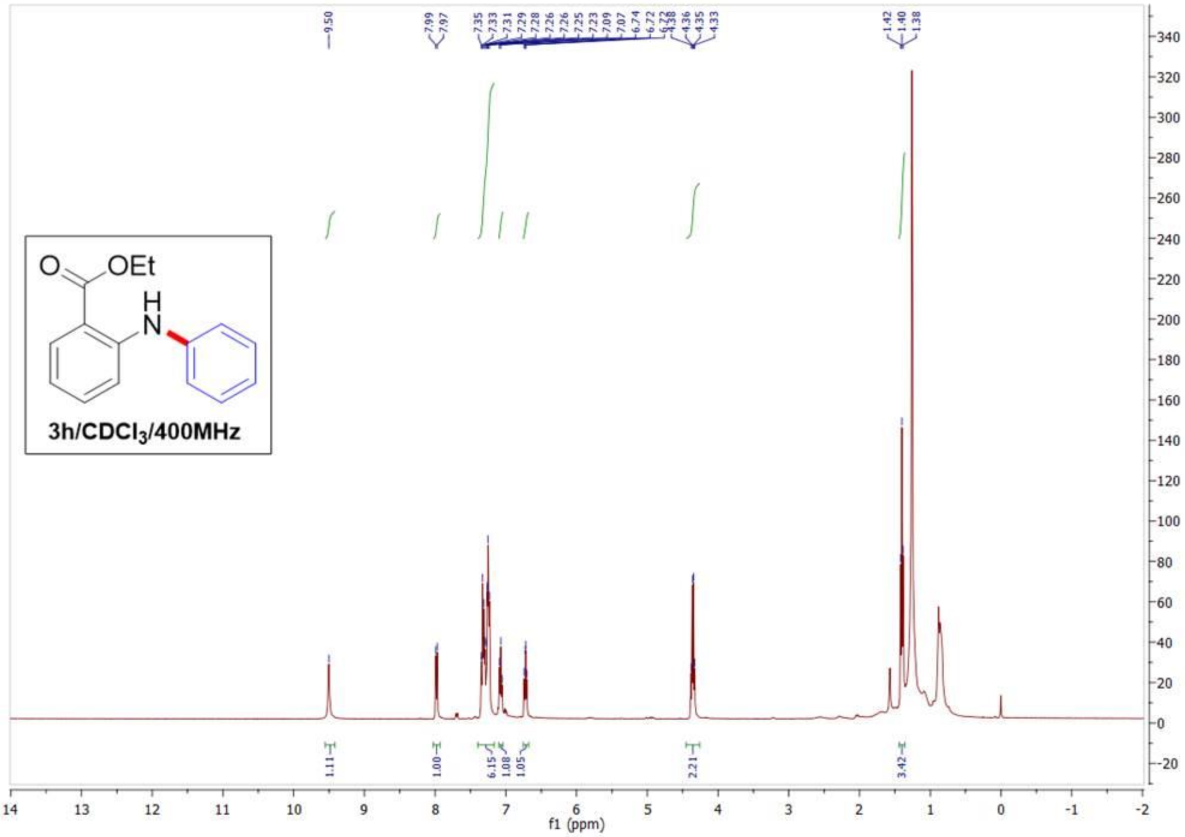
3h/CDCl3/400MHz
f1 (ppm)

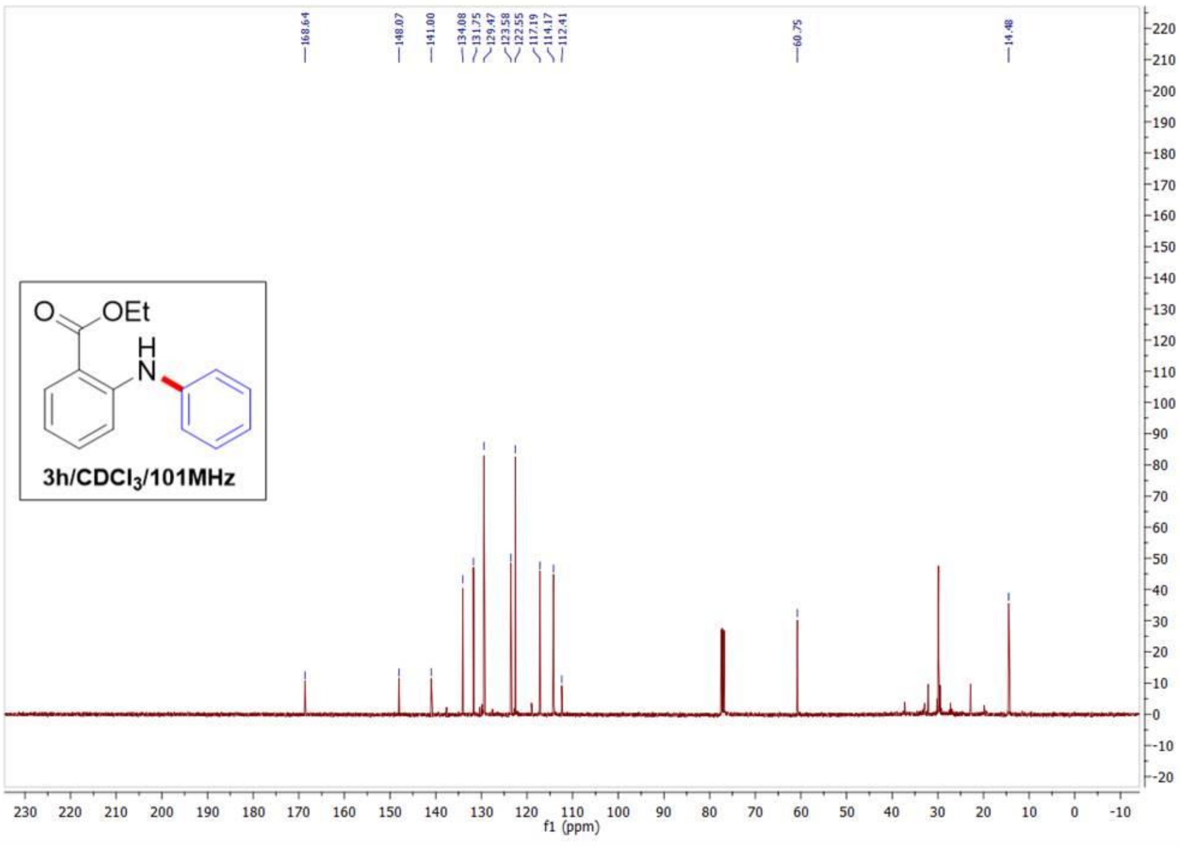
3h/CDCl3/101MHz
f1 (ppm)

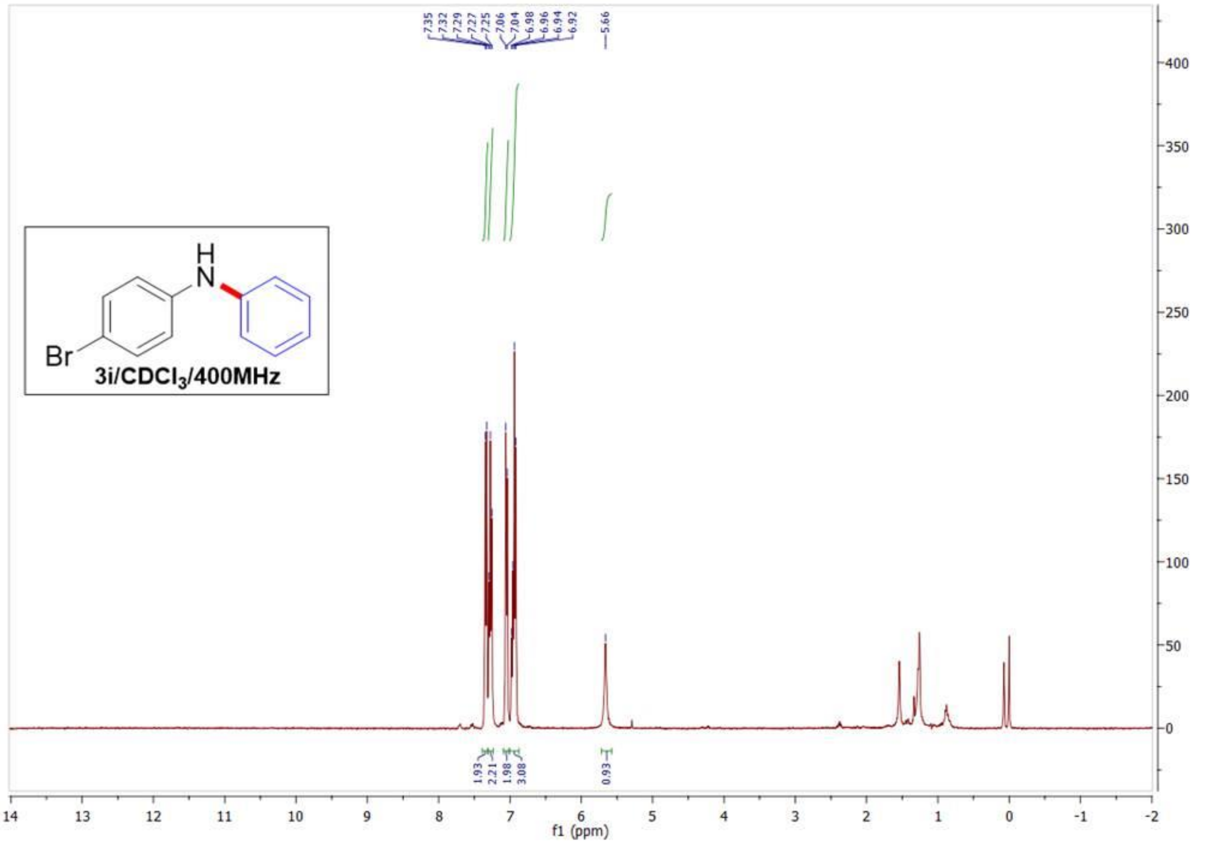
3i/CDCl3/400MHz
Br
H
N
7.35
7.32
7.29
7.27
7.25
7.06
7.04
6.98
6.96
6.94
6.92
5.66
1.93
2.21
1.98
3.08
0.93
f1 (ppm)

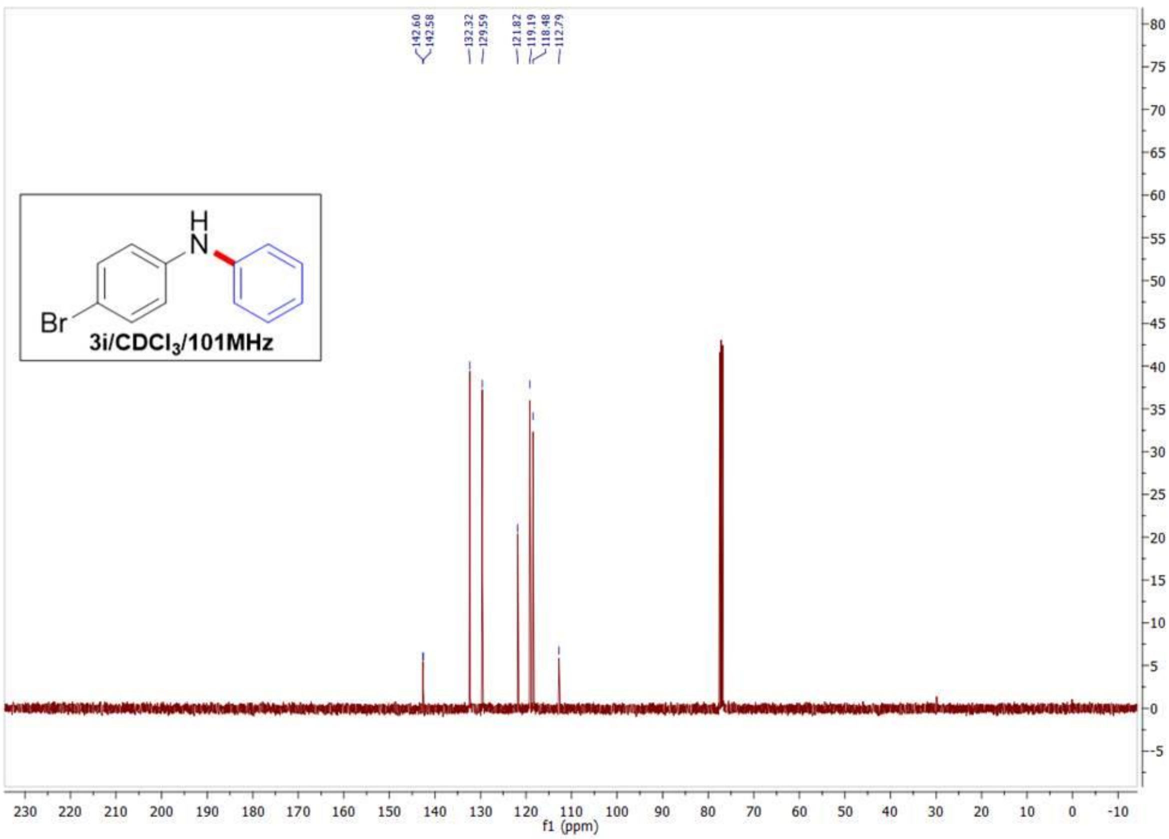
3i/CDCl3/101MHz
Br
H
N
142.60
142.58
132.32
129.59
121.82
119.19
118.48
112.79
f1 (ppm)

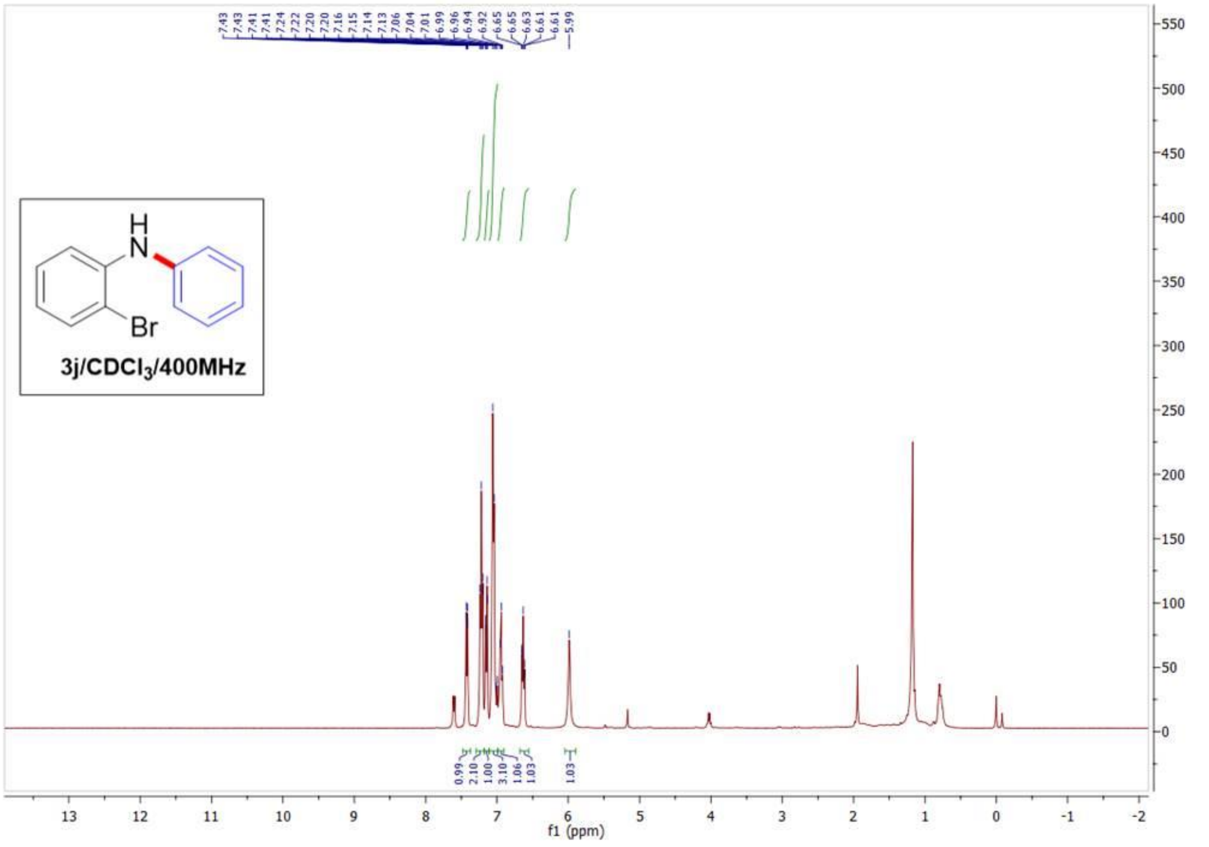
3j/CDCl3/400MHz
f1 (ppm)

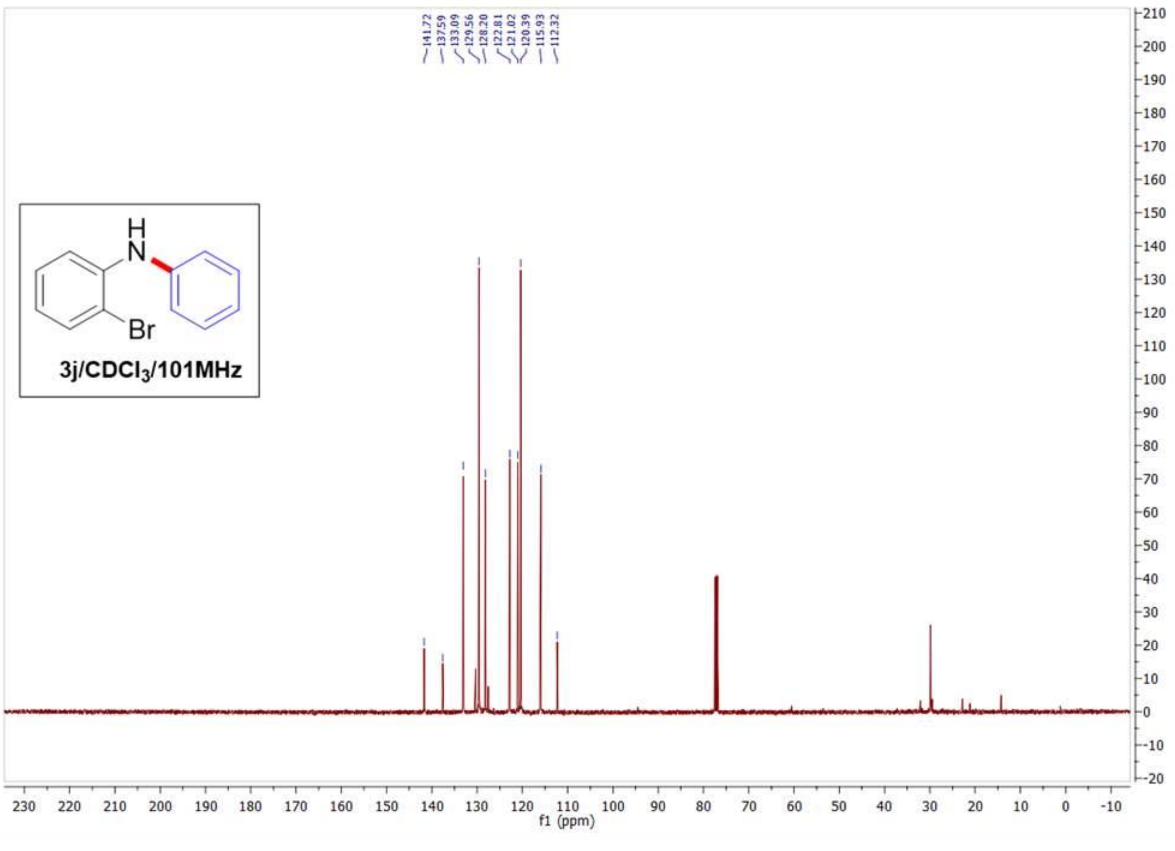
3j/CDCl3/101MHz
f1 (ppm)

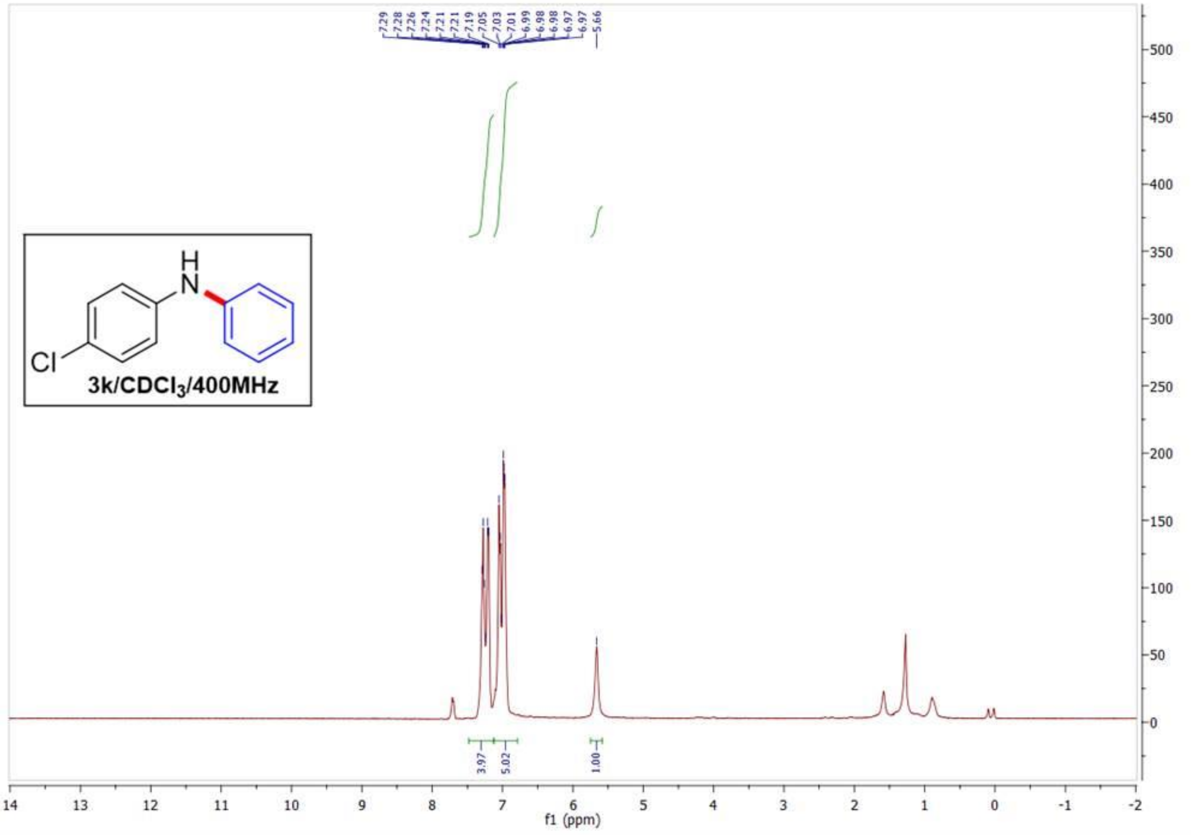
7.29
7.28
7.26
7.24
7.21
7.21
7.19
7.05
7.03
7.01
6.99
6.98
6.98
6.97
6.97
5.66
H
N
Cl
3k/CDCl3/400MHz
3.97
5.02
1.00
f1 (ppm)

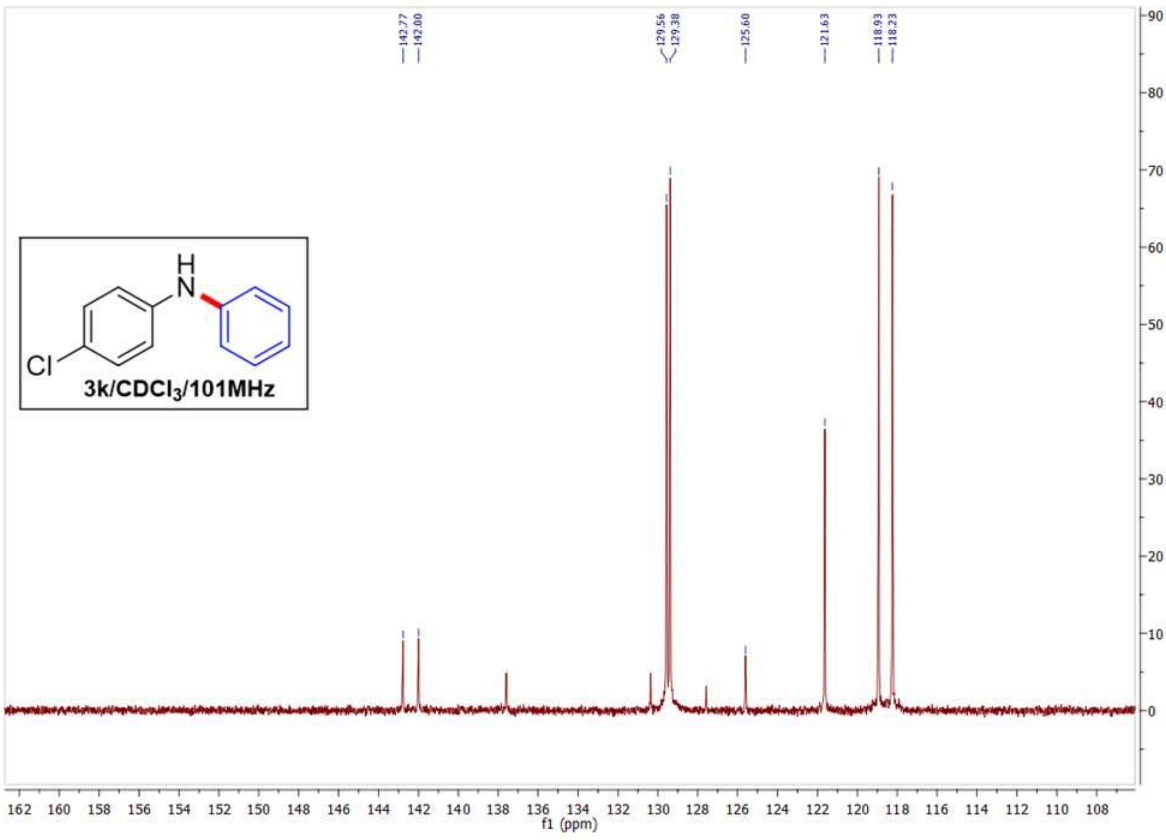
142.77
142.00
129.56
129.38
125.60
121.63
118.93
118.23
H
N
Cl
3k/CDCl3/101MHz
f1 (ppm)

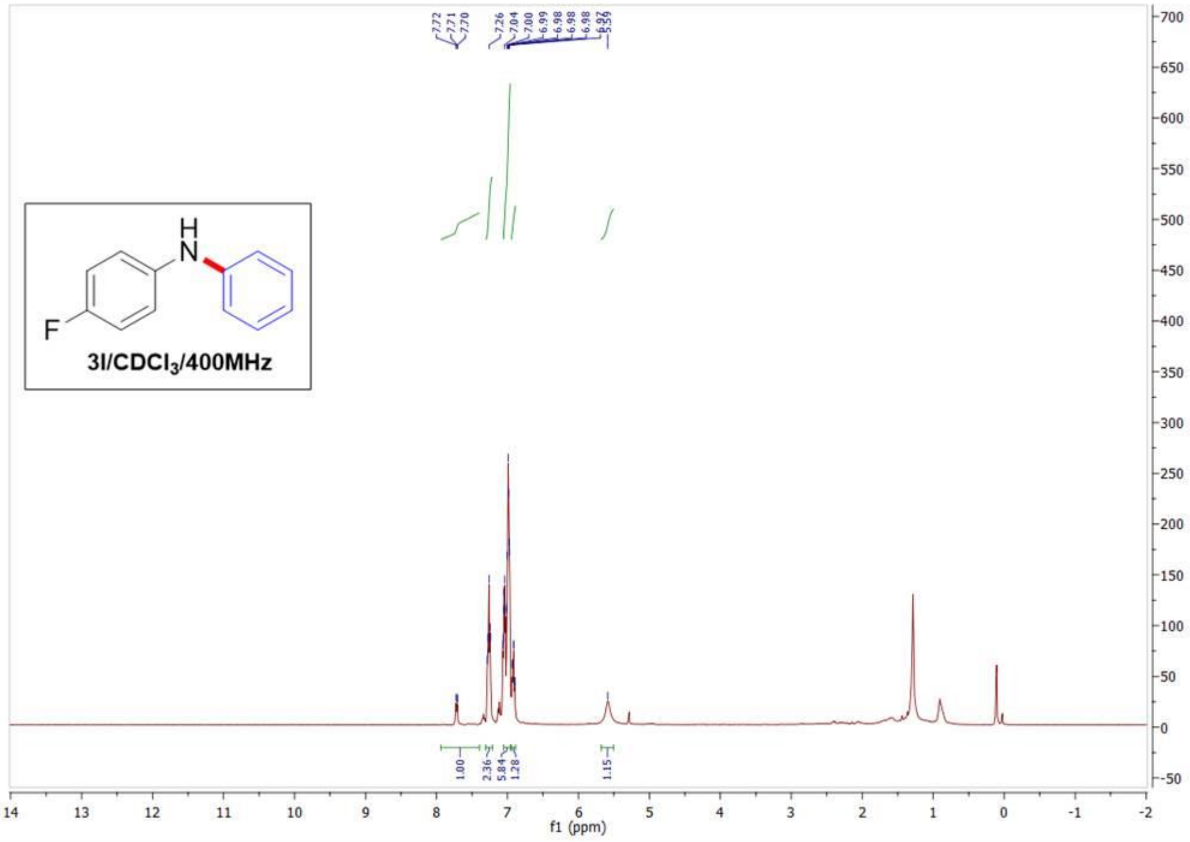
3l/CDCl3/400MHz
f1 (ppm)

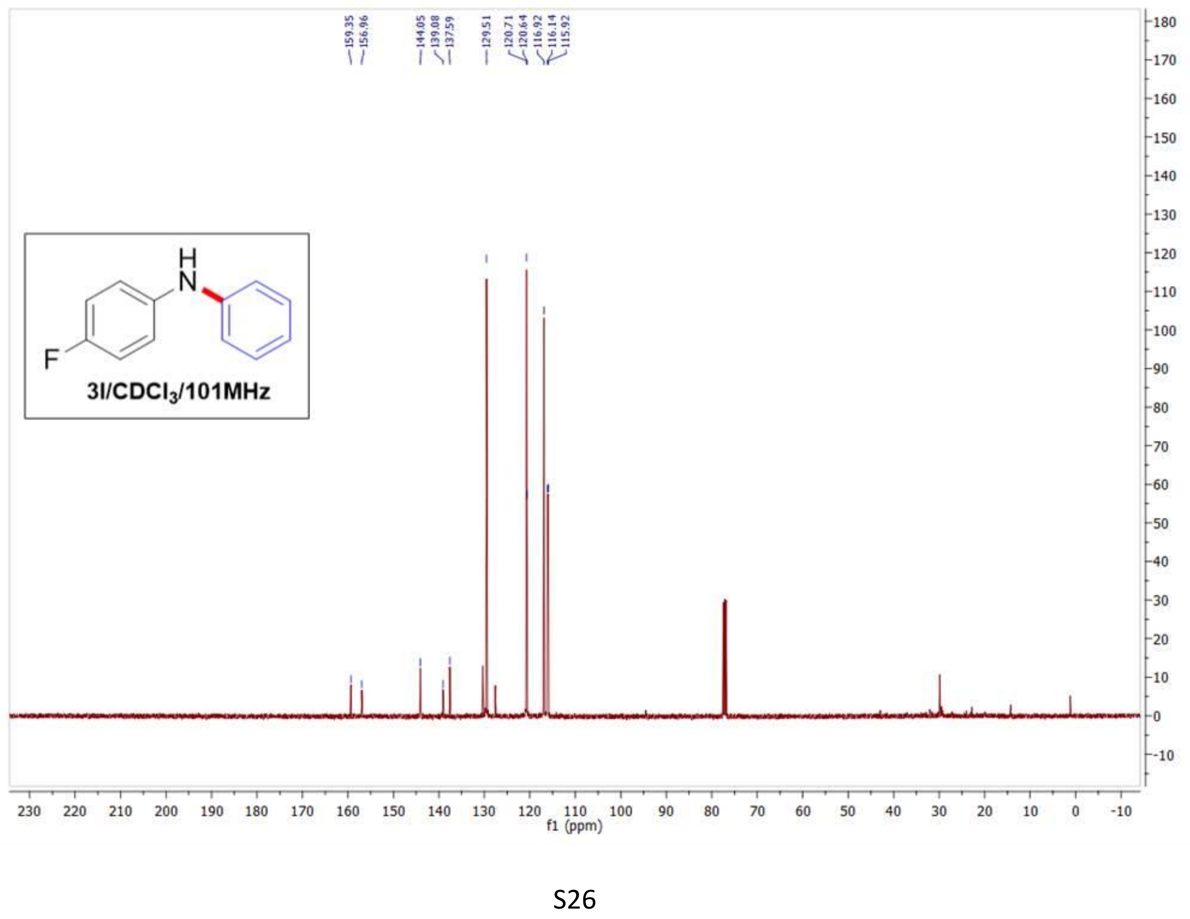
3l/CDCl3/101MHz
f1 (ppm)

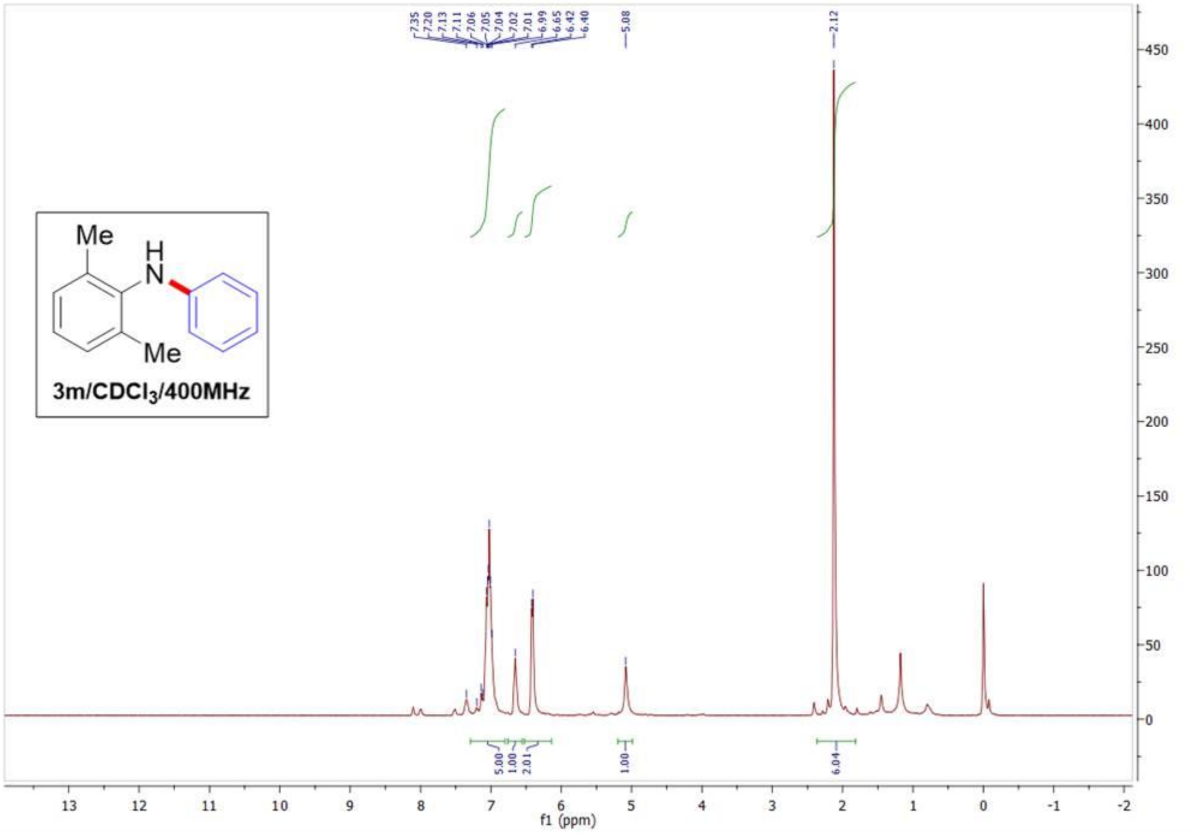
7.35
7.20
7.13
7.11
7.06
7.05
7.04
7.02
7.01
6.99
6.65
6.42
6.40
5.08
2.12
Me
H
N
Me
3m/CDCl3/400MHz
5.00
1.00
2.01
1.00
6.04
f1 (ppm)

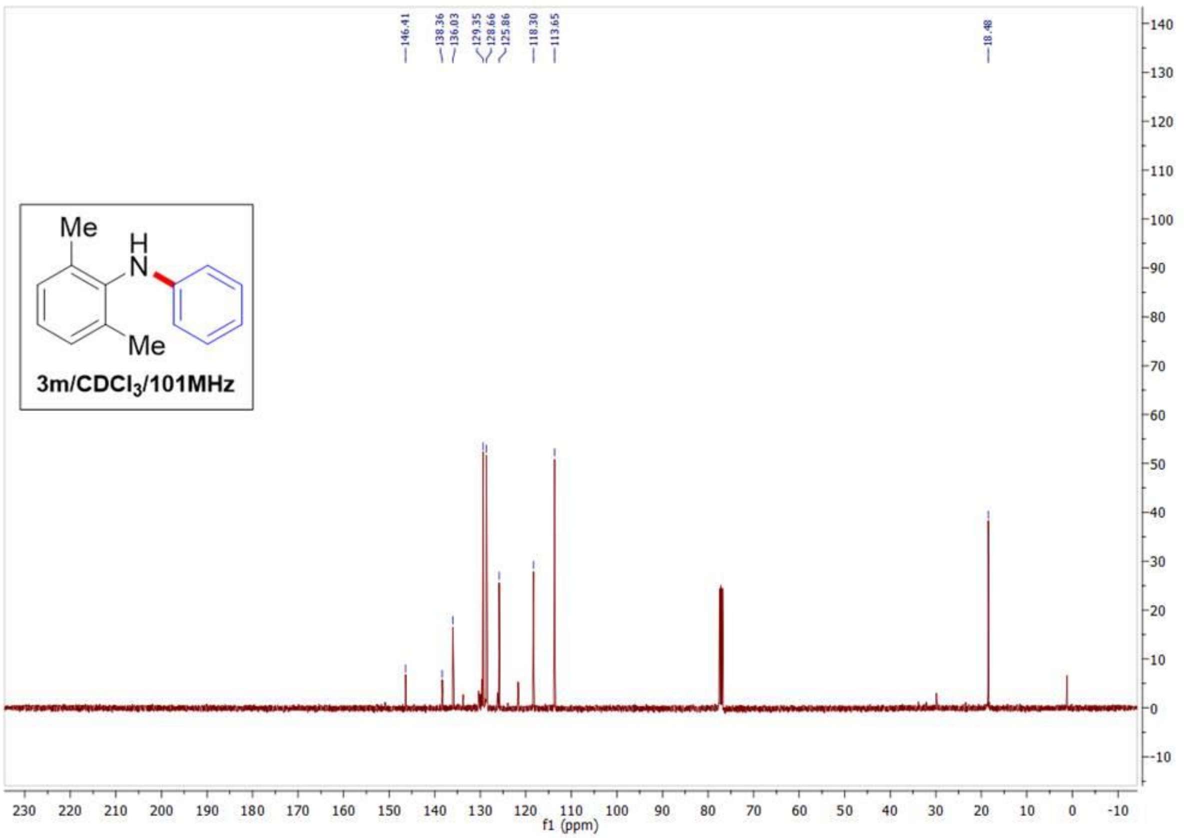
146.41
138.36
136.03
129.35
128.66
125.86
118.30
113.65
18.48
Me
H
N
Me
3m/CDCl3/101MHz
f1 (ppm)

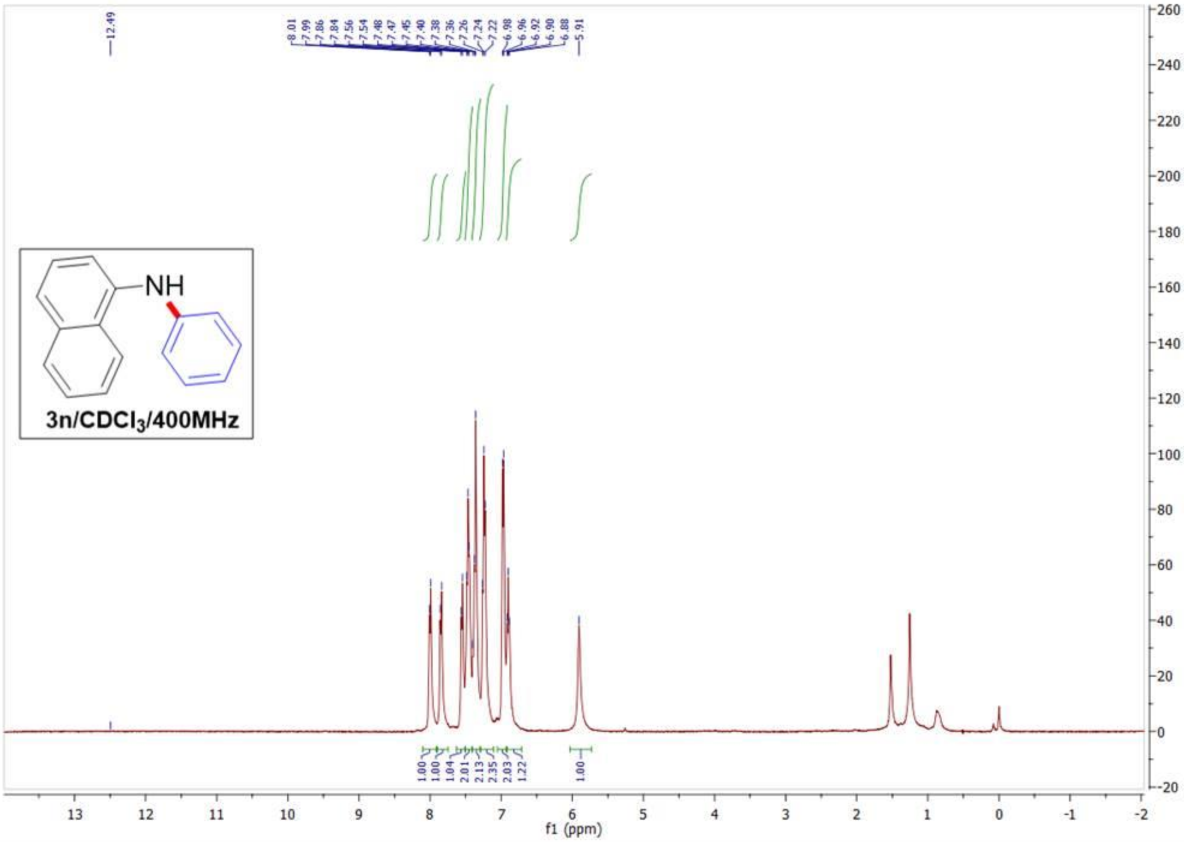
12.49
8.01
7.99
7.86
7.84
7.56
7.54
7.48
7.47
7.45
7.40
7.38
7.36
7.26
7.24
7.22
6.98
6.96
6.92
6.90
6.88
5.91
NH
3n/CDCl3/400MHz
1.00
1.00
1.04
2.01
2.13
2.35
2.03
1.22
1.00
f1 (ppm)

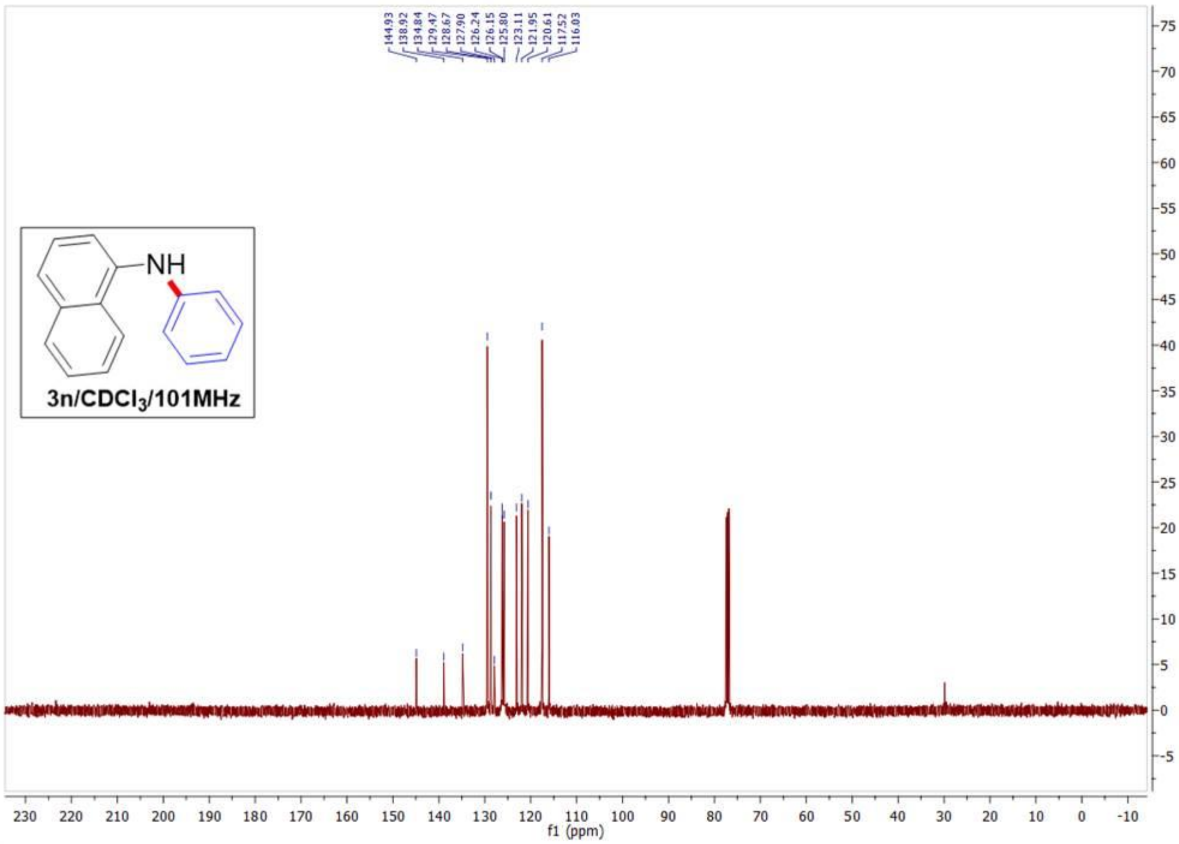
144.93
138.92
134.84
129.47
128.67
127.90
126.24
126.15
125.80
123.11
121.95
120.61
117.52
116.03
NH
3n/CDCl3/101MHz
f1 (ppm)

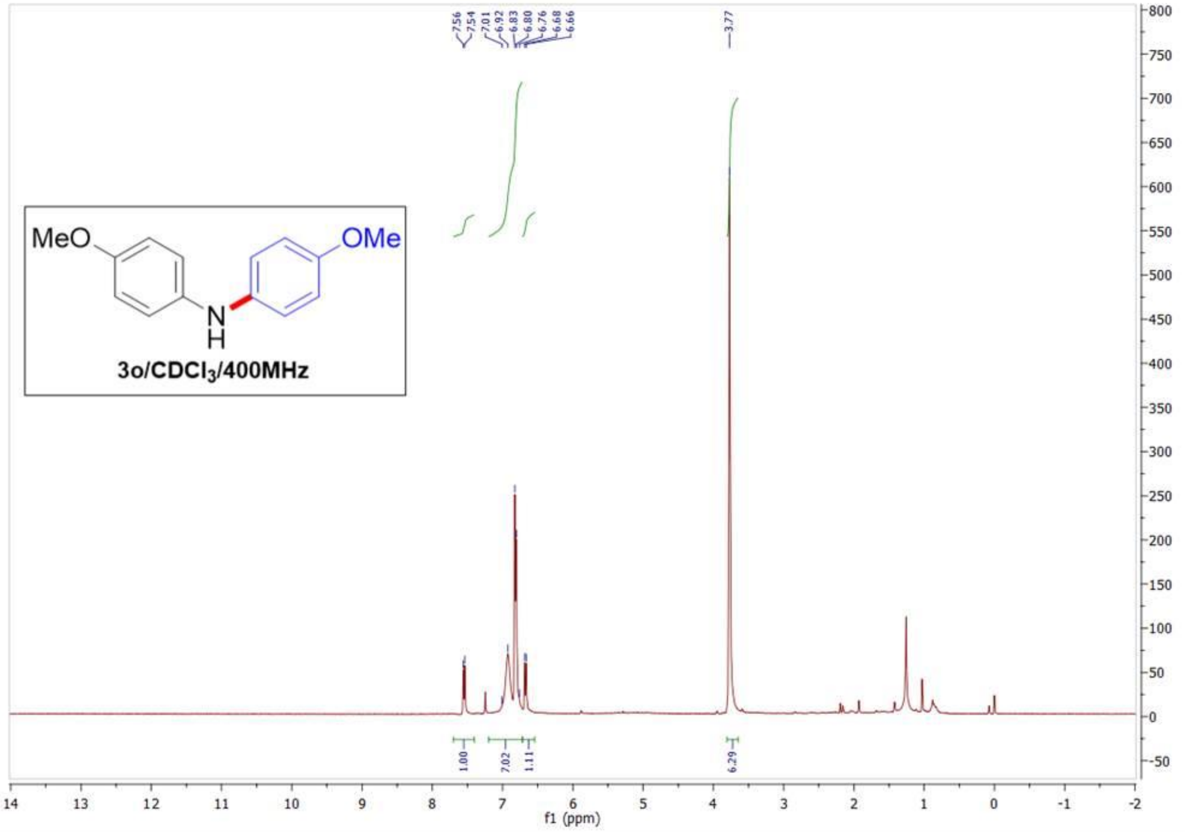
7.56
7.54
7.01
6.92
6.83
6.80
6.76
6.68
6.66
3.77
MeO
OMe
N
H
3o/CDCl3/400MHz
1.00
7.02
1.11
6.29
f1 (ppm)

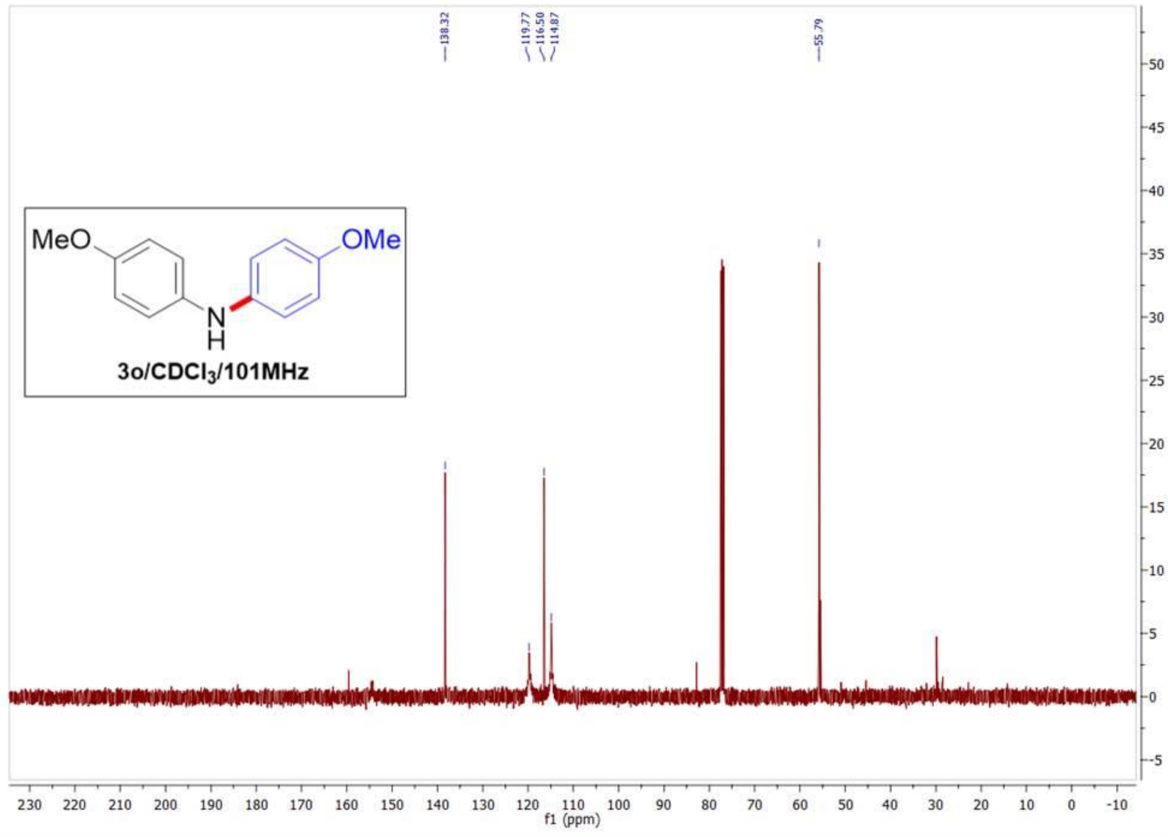
138.32
119.77
116.50
114.87
55.79
MeO
OMe
N
H
3o/CDCl3/101MHz
f1 (ppm)

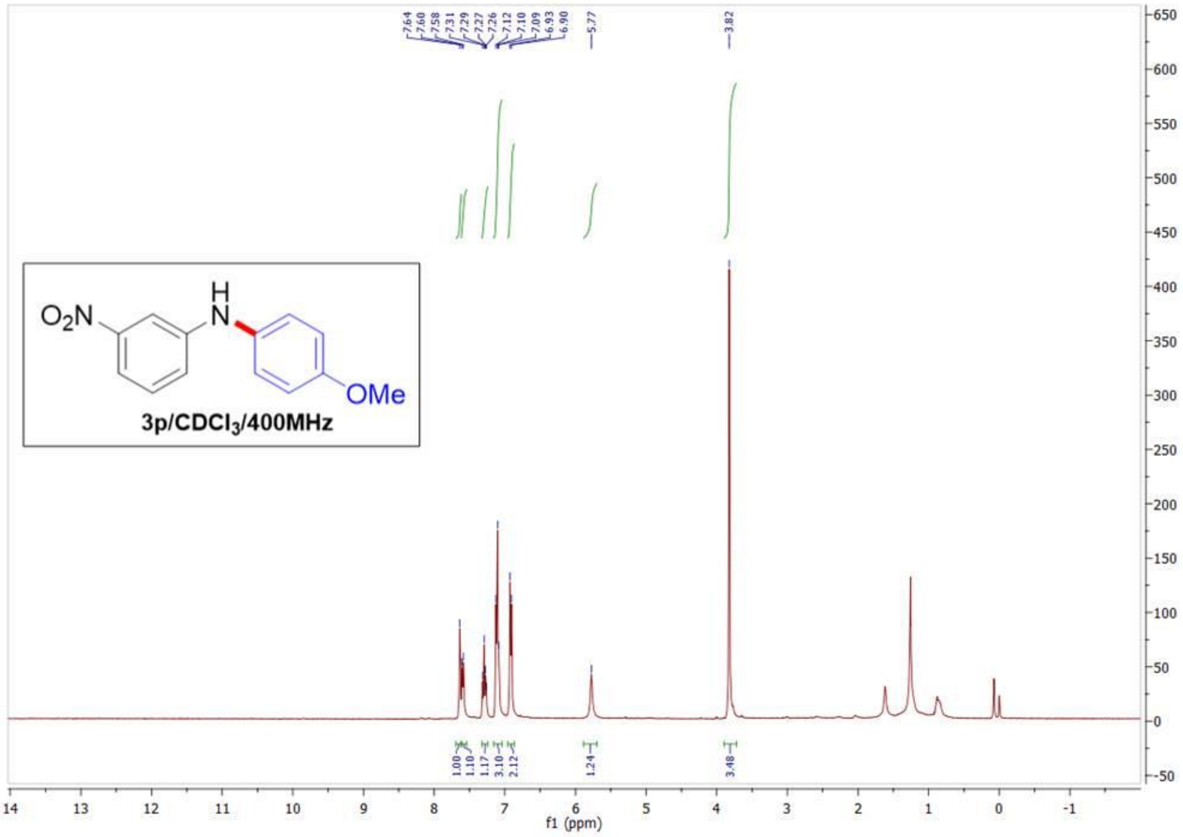
3p/CDCl3/400MHz
f1 (ppm)

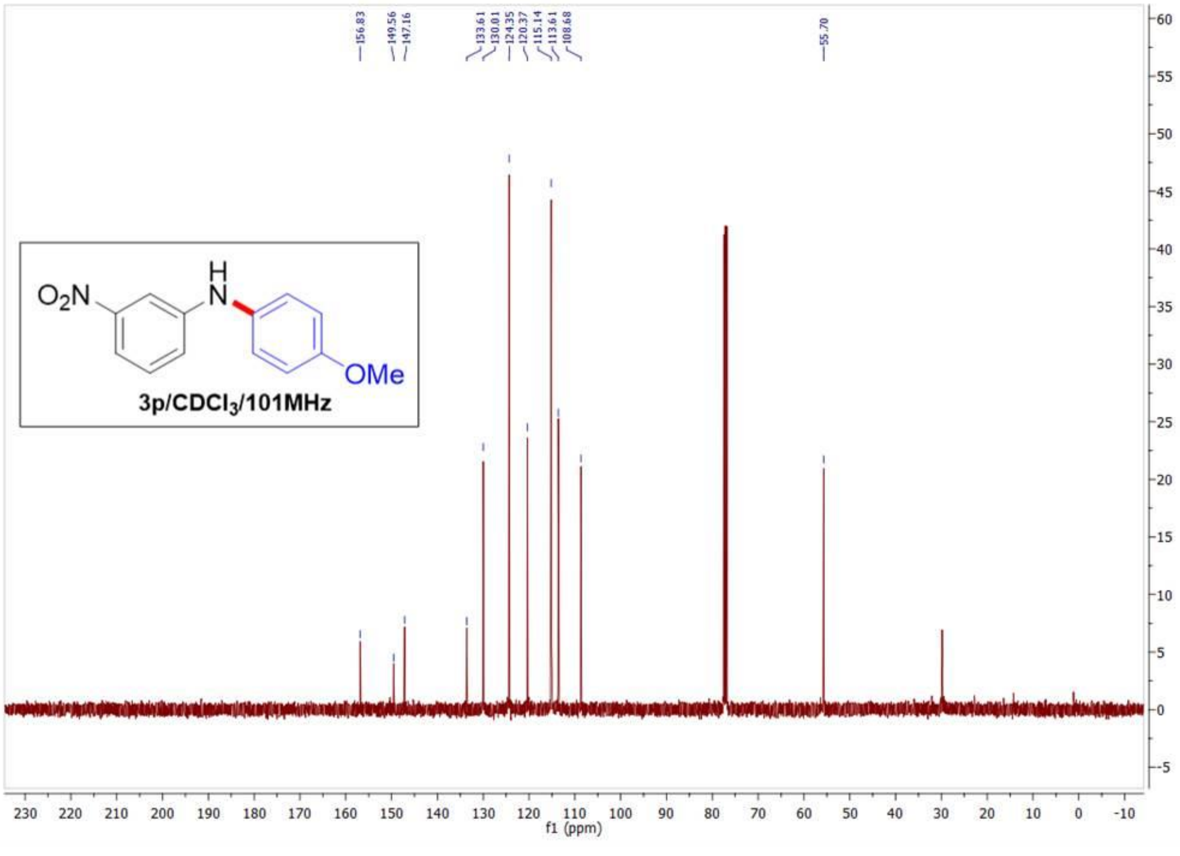
3p/CDCl3/101MHz
f1 (ppm)

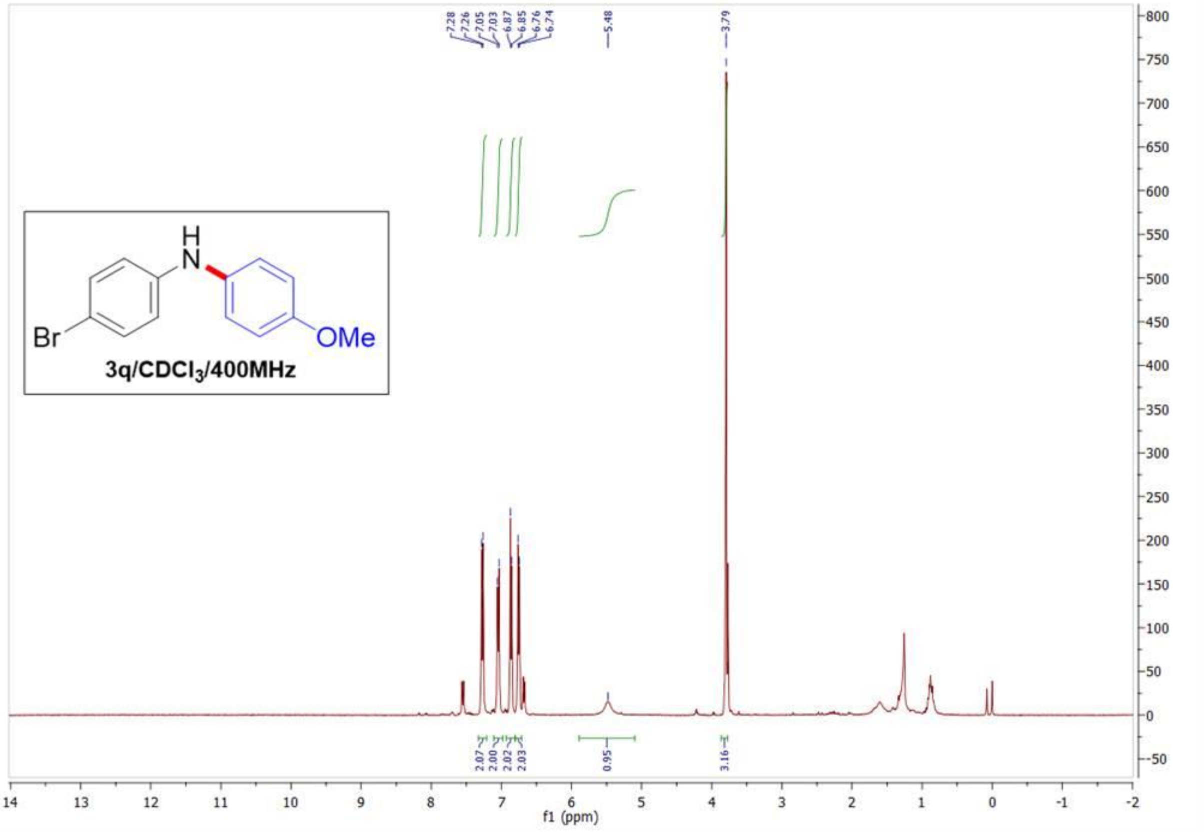

7.28
7.26
7.05
7.03
6.87
6.85
6.76
6.74
5.48
3.79
Br
OMe
3q/CDCl$_3$/400MHz
2.07
2.00
2.02
2.03
0.95
3.16
f1 (ppm)

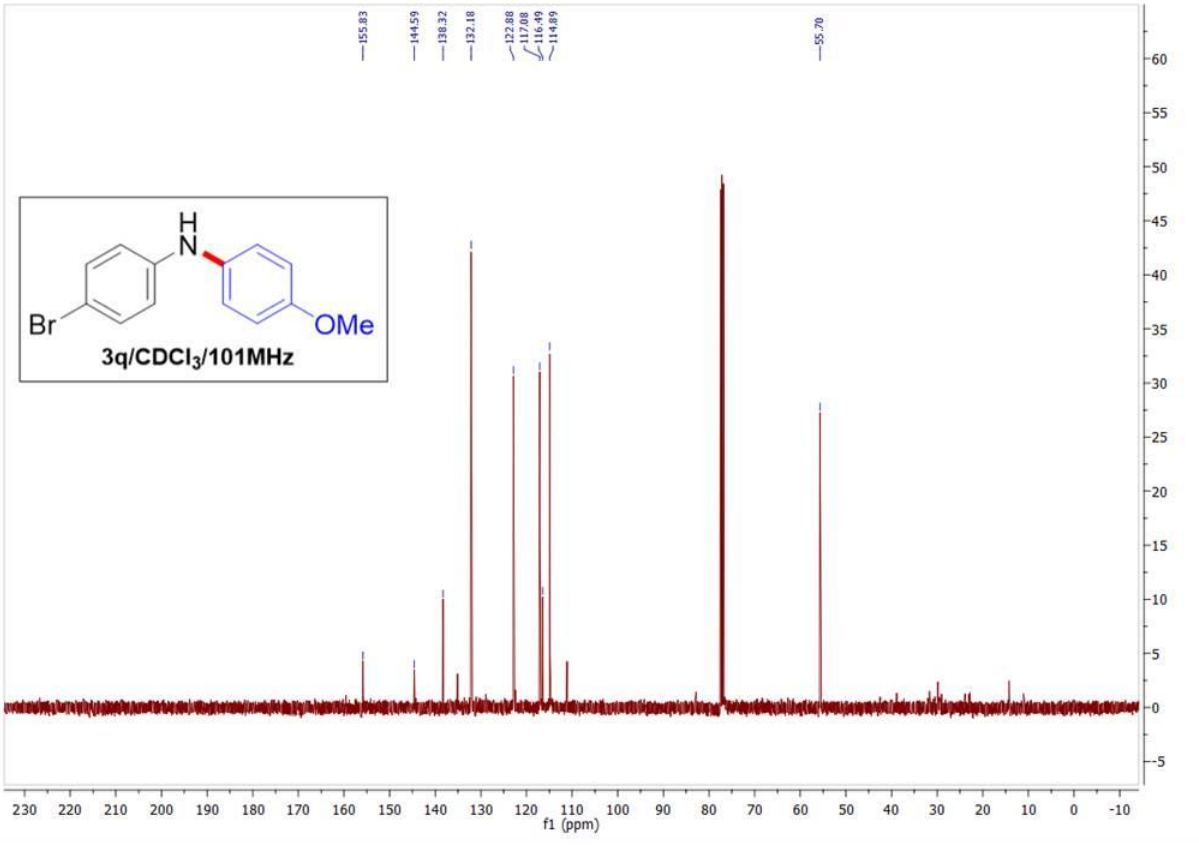

155.83
144.59
138.32
132.18
122.88
117.08
116.49
114.89
55.70
Br
OMe
3q/CDCl$_3$/101MHz
f1 (ppm)

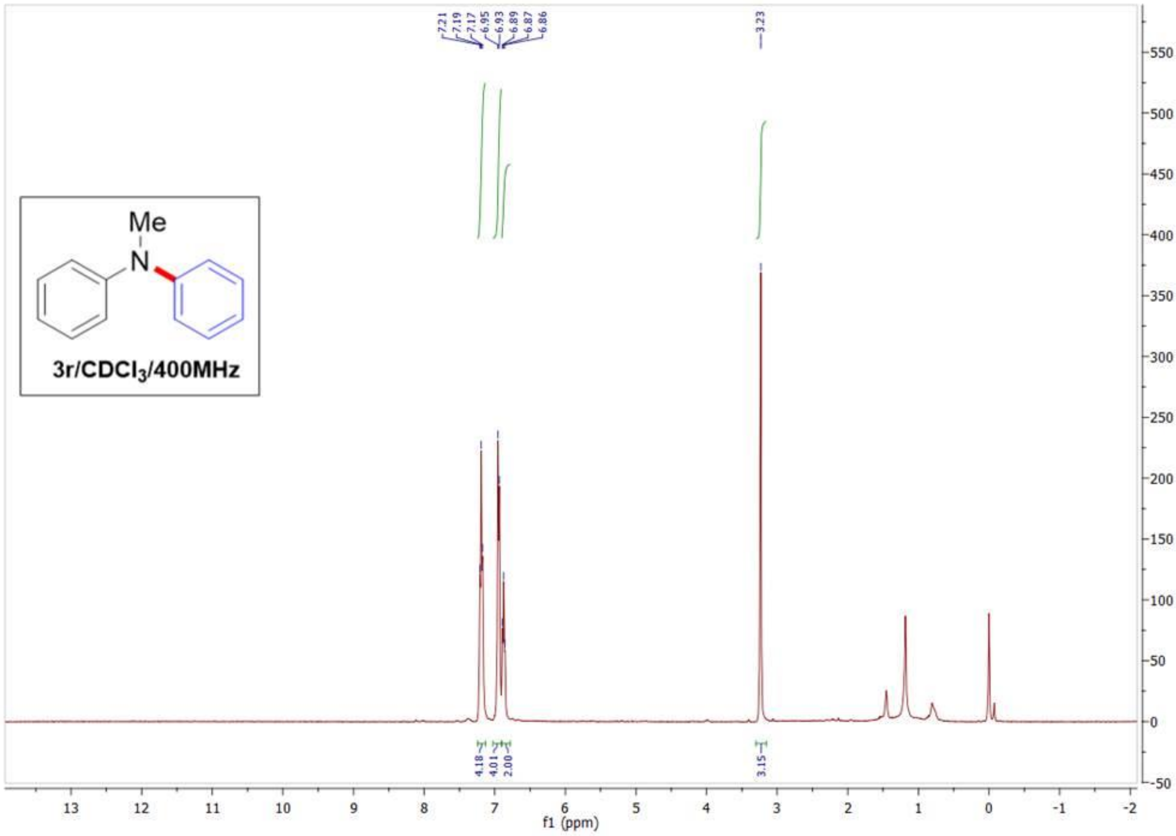
7.21
7.19
7.17
6.95
6.93
6.93
6.89
6.87
6.86
3.23
Me
N
3r/CDCl$_3$/400MHz
4.18
4.01
2.00
3.15
f1 (ppm)

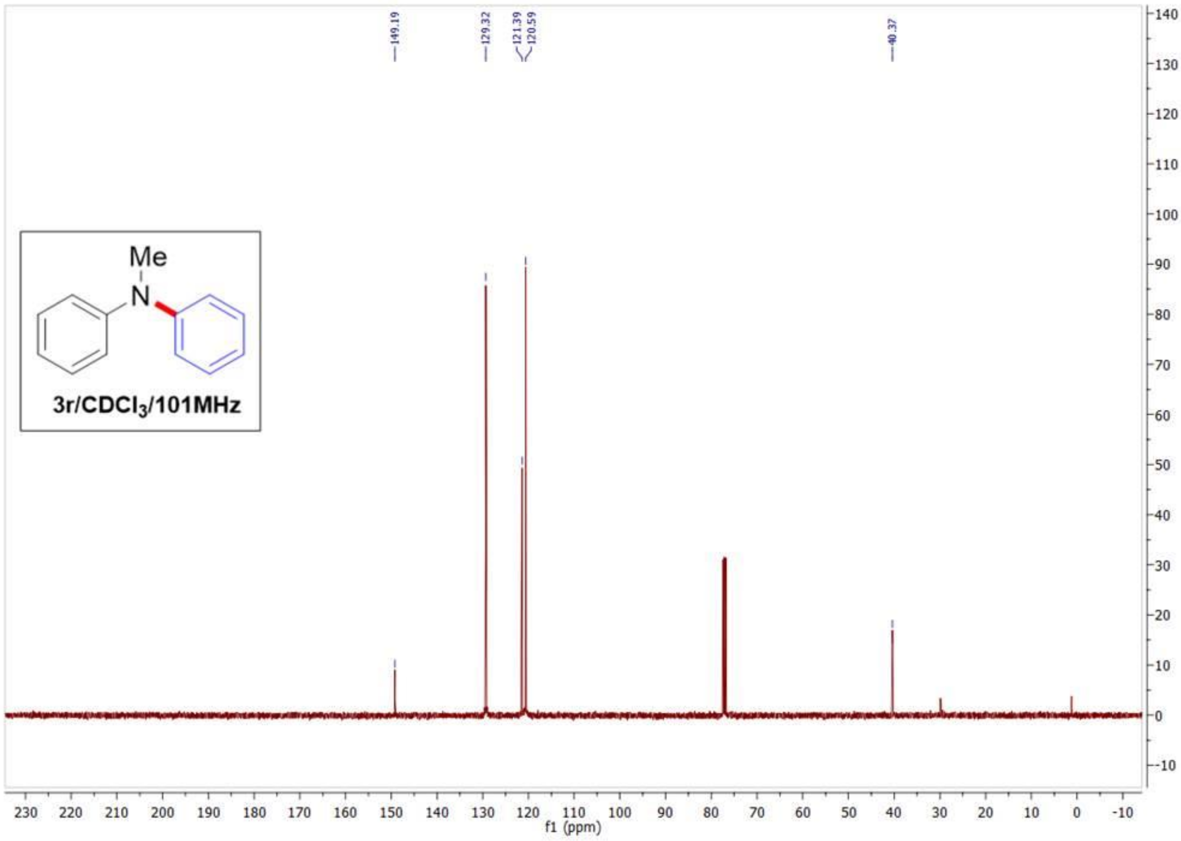
149.19
129.32
121.39
120.59
40.37
Me
N
3r/CDCl$_3$/101MHz
f1 (ppm)

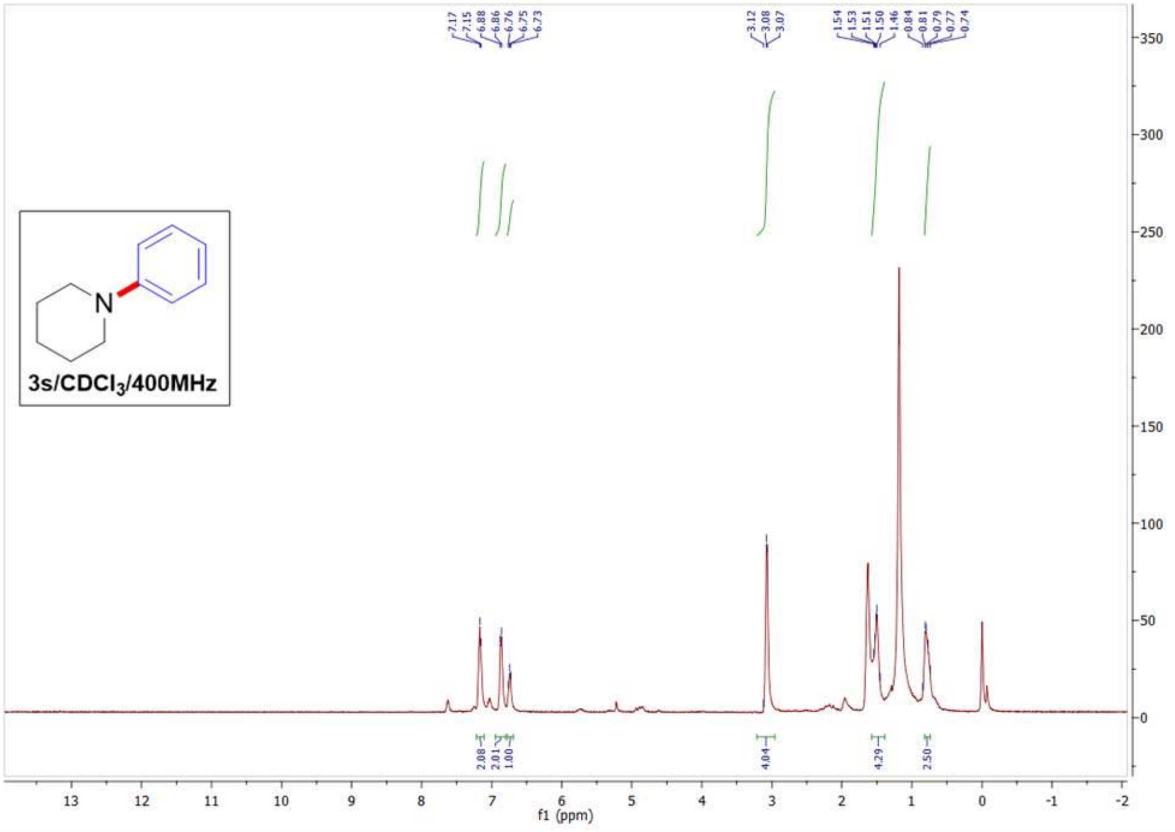
3s/CDCl3/400MHz
f1 (ppm)

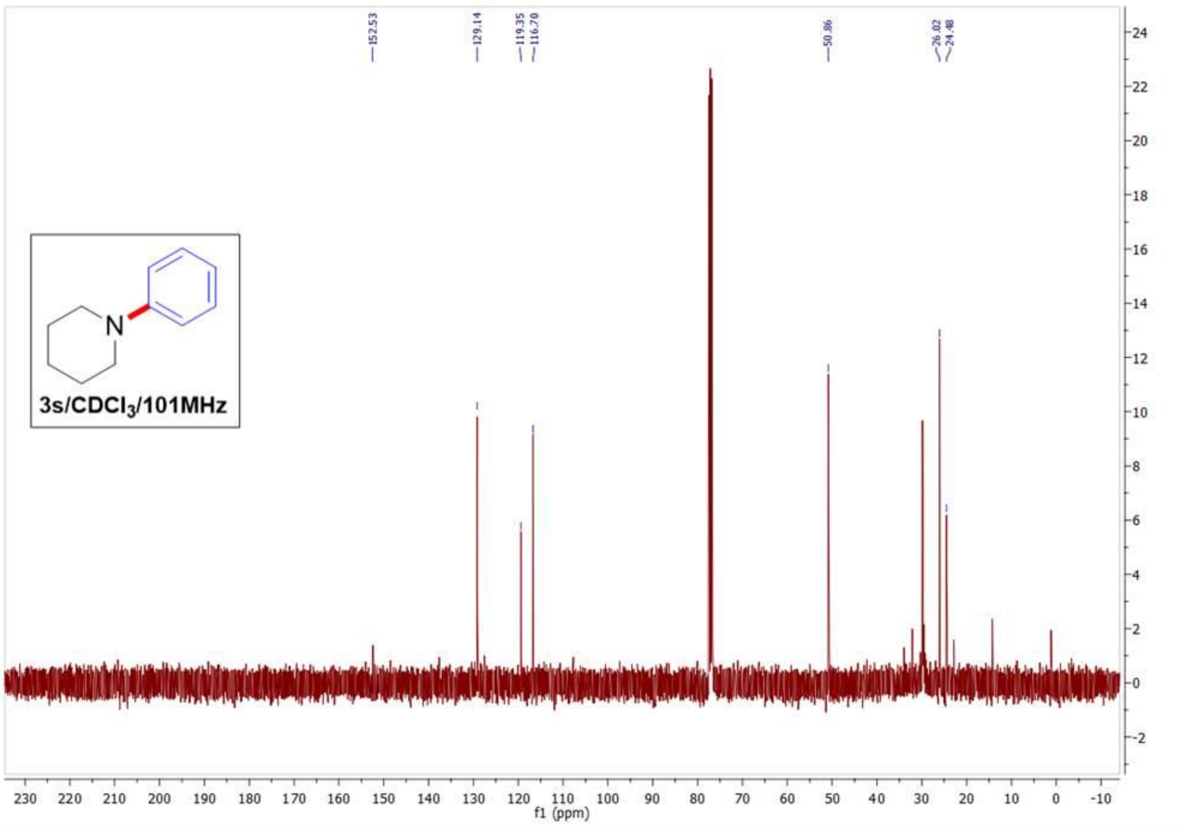
3s/CDCl3/101MHz
f1 (ppm)

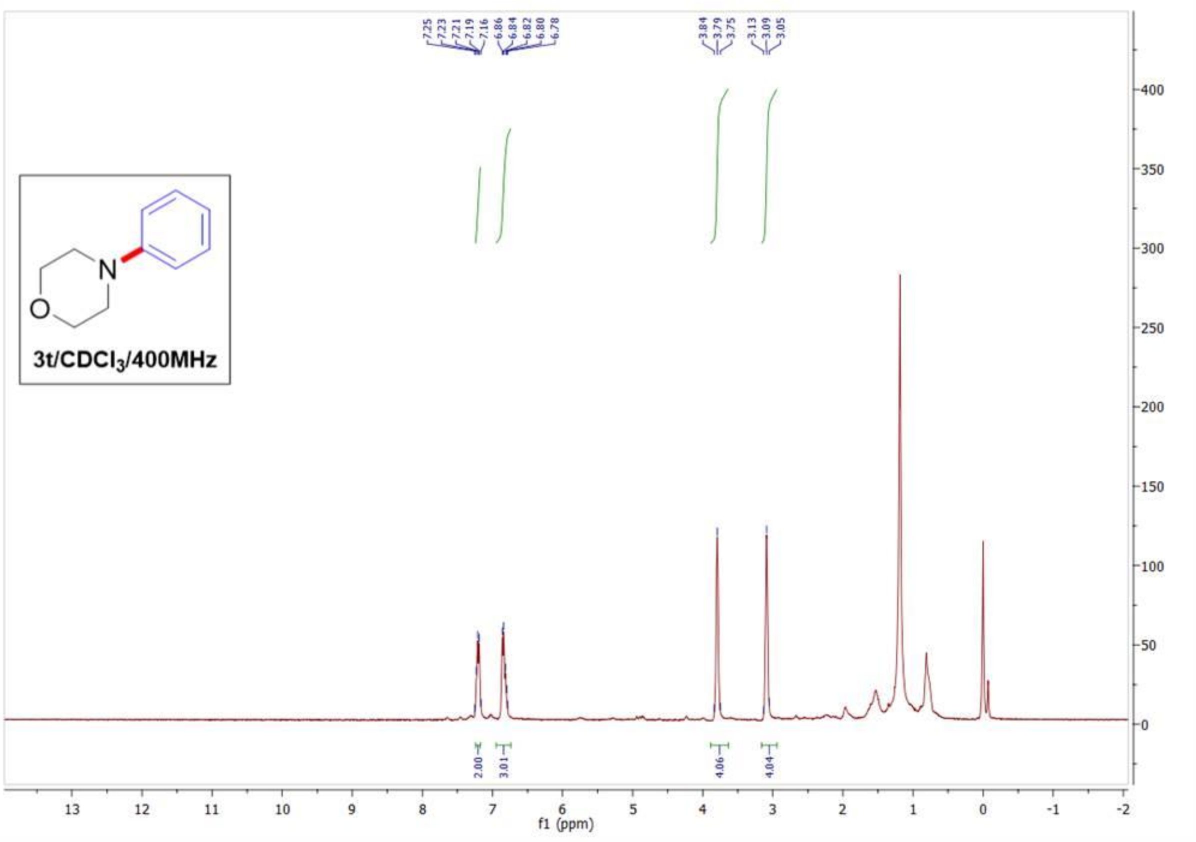
7.25
7.23
7.21
7.19
7.16
6.86
6.84
6.82
6.80
6.78
3.84
3.79
3.75
3.13
3.09
3.05
N
O
3t/CDCl3/400MHz
2.00
3.01
4.06
4.04
f1 (ppm)

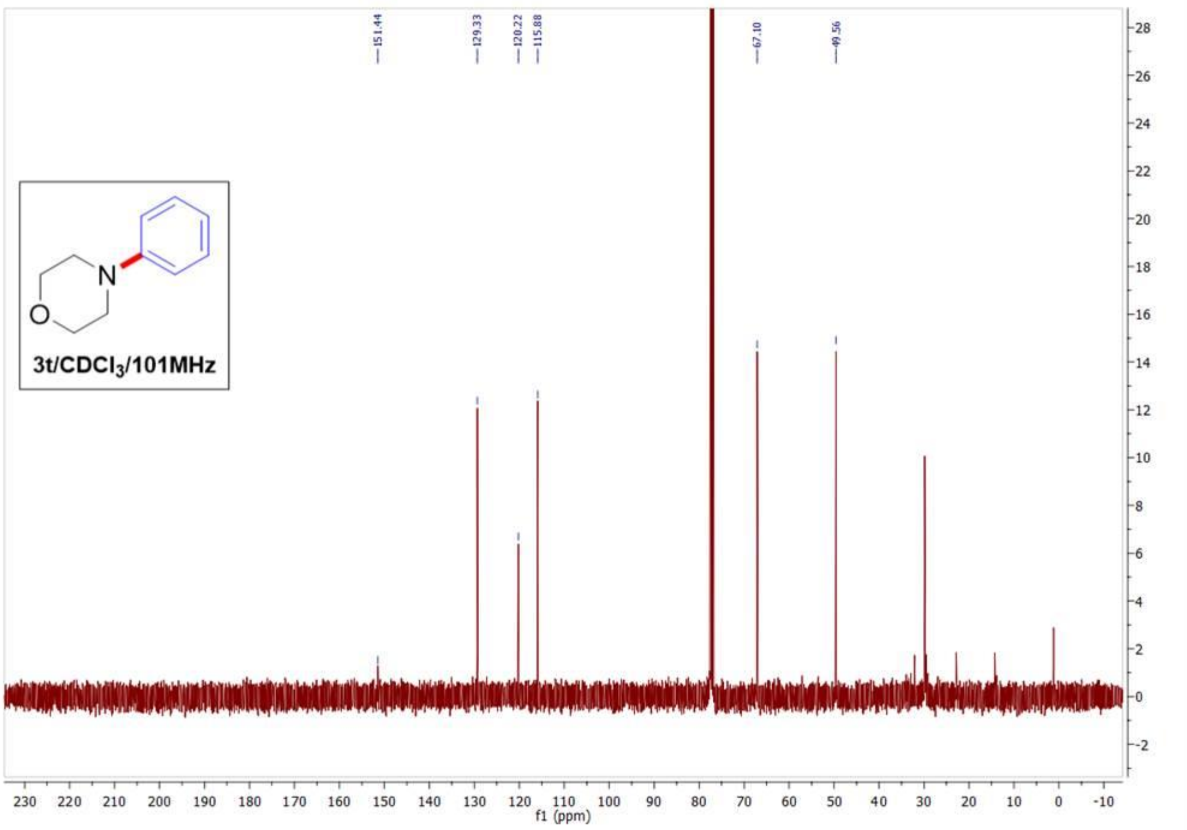
151.44
129.33
120.22
115.88
67.10
49.56
N
O
3t/CDCl3/101MHz
f1 (ppm)

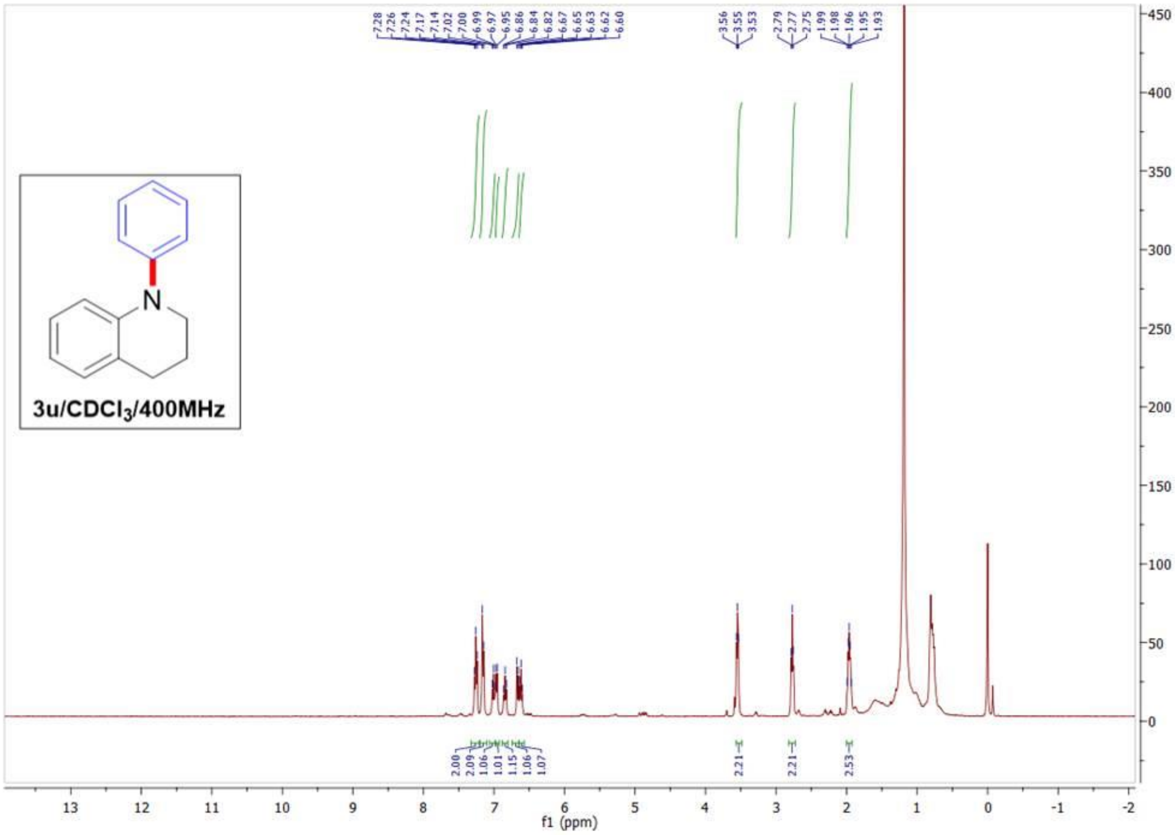
3u/CDCl$_3$/400MHz
f1 (ppm)

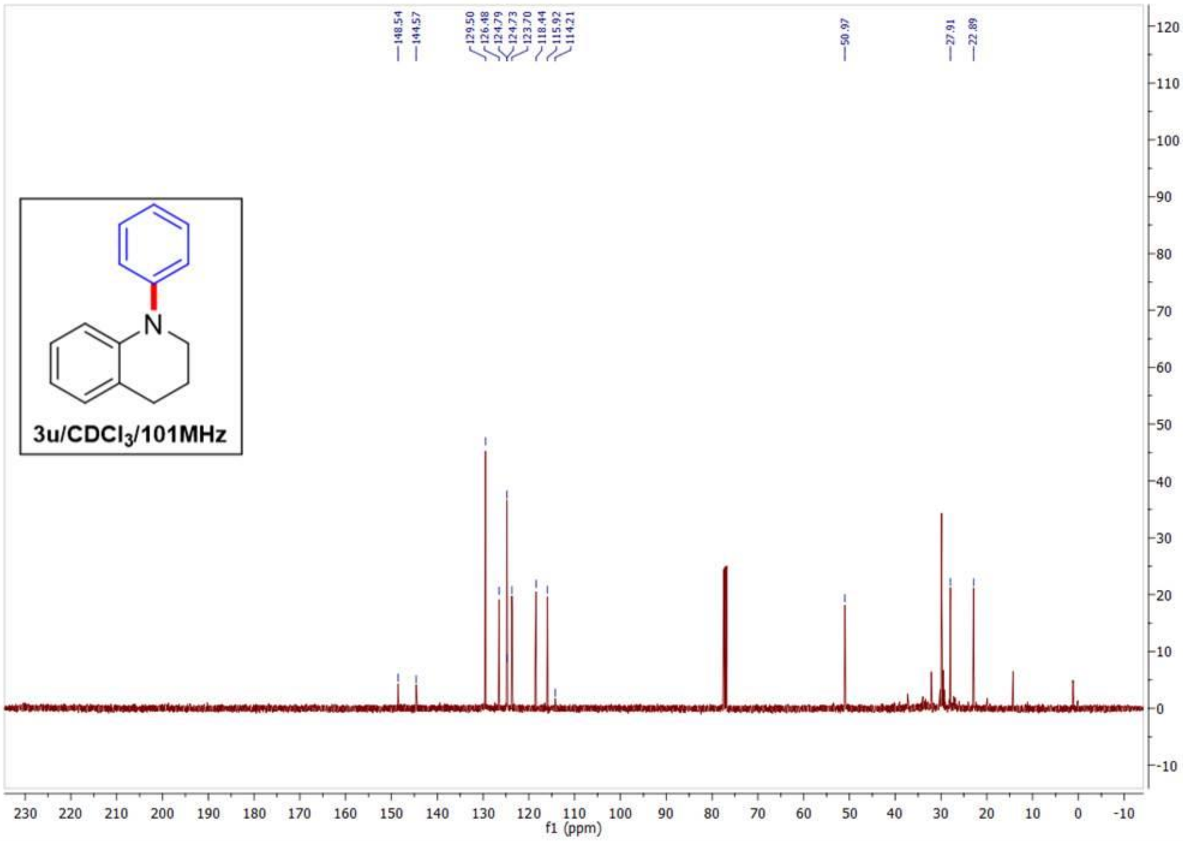
3u/CDCl$_3$/101MHz
f1 (ppm)

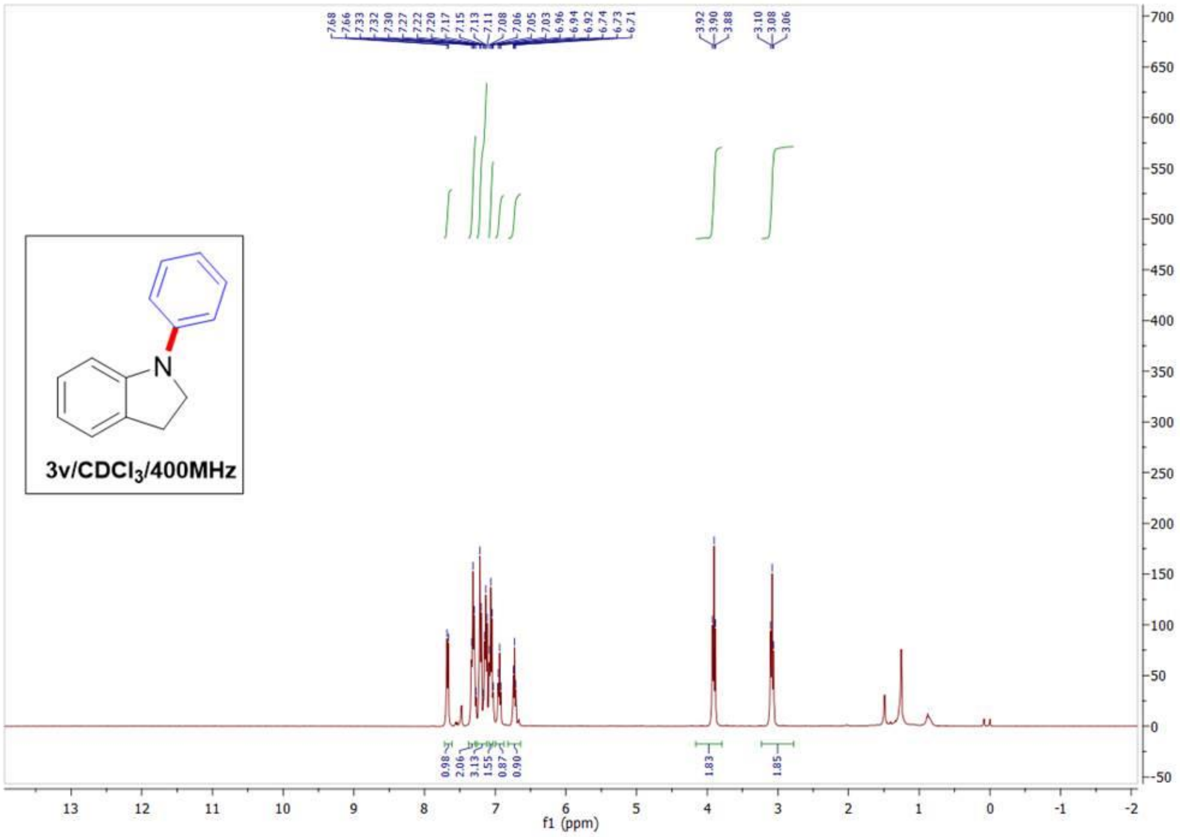

7.68
7.66
7.33
7.32
7.30
7.27
7.22
7.20
7.17
7.15
7.13
7.11
7.08
7.06
7.05
7.03
6.96
6.94
6.92
6.74
6.73
6.71
3.92
3.90
3.88
3.10
3.08
3.06
0.98
2.06
3.13
1.55
0.87
0.90
1.83
1.85
N
3v/CDCl3/400MHz
f1 (ppm)

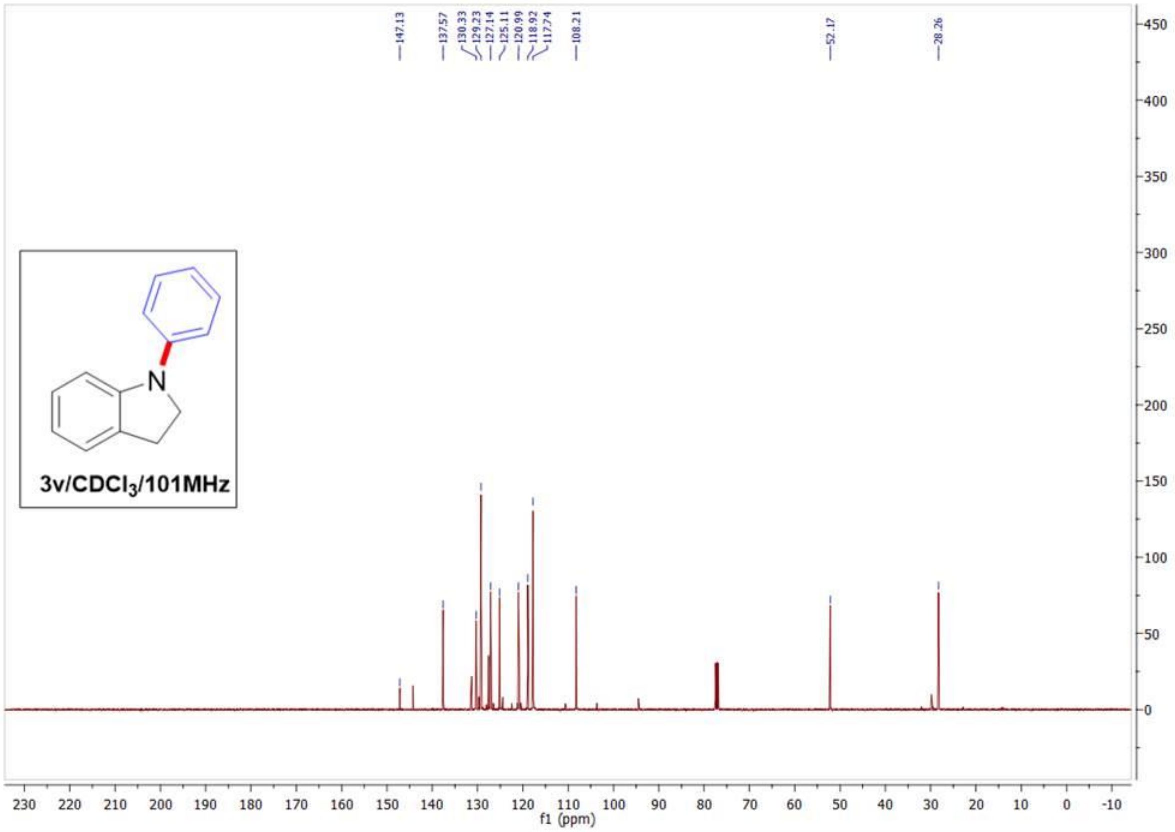

147.13
137.57
130.33
129.23
127.14
125.11
120.99
118.92
117.74
108.21
52.17
28.26
N
3v/CDCl3/101MHz
f1 (ppm)

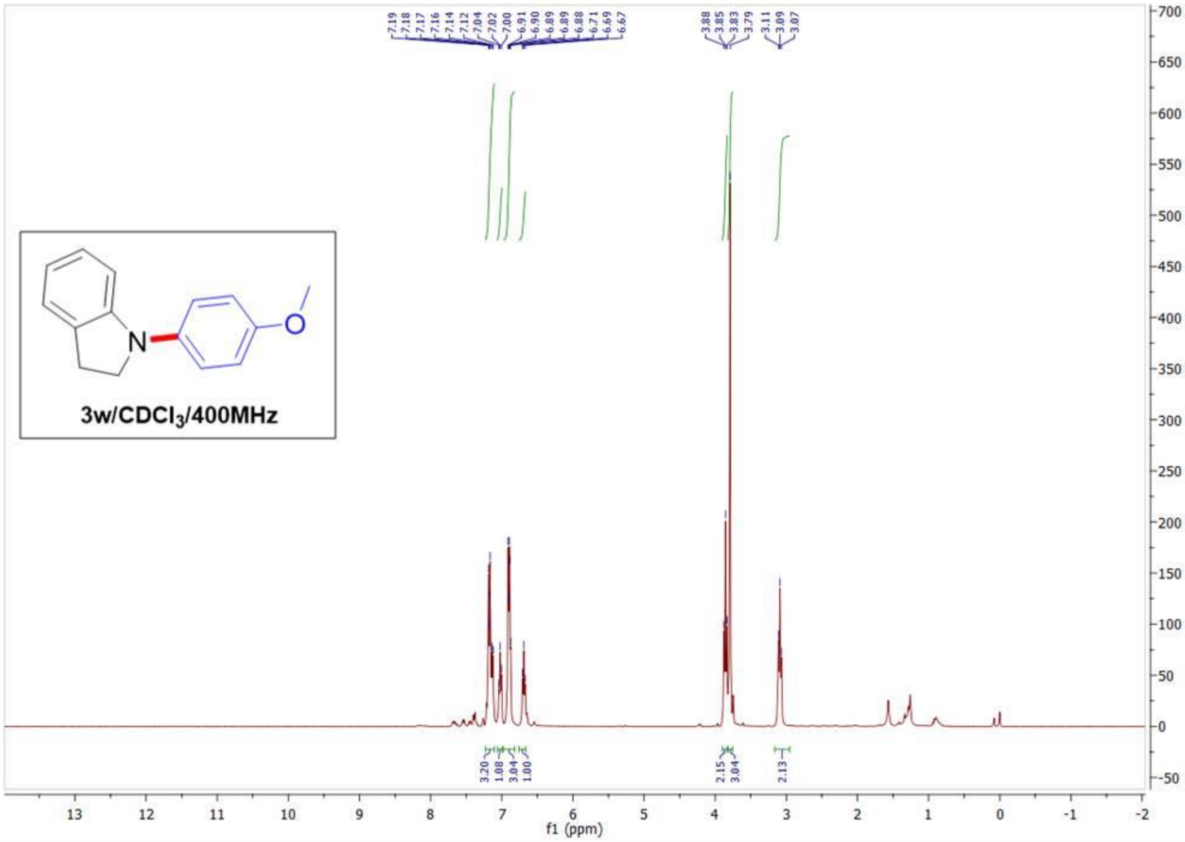
7.19
7.18
7.17
7.16
7.14
7.12
7.04
7.02
7.00
6.91
6.90
6.89
6.89
6.88
6.71
6.69
6.67
3.88
3.85
3.83
3.79
3.11
3.09
3.07
3w/CDCl3/400MHz
3.20
1.08
3.04
1.00
2.15
3.04
2.13
f1 (ppm)

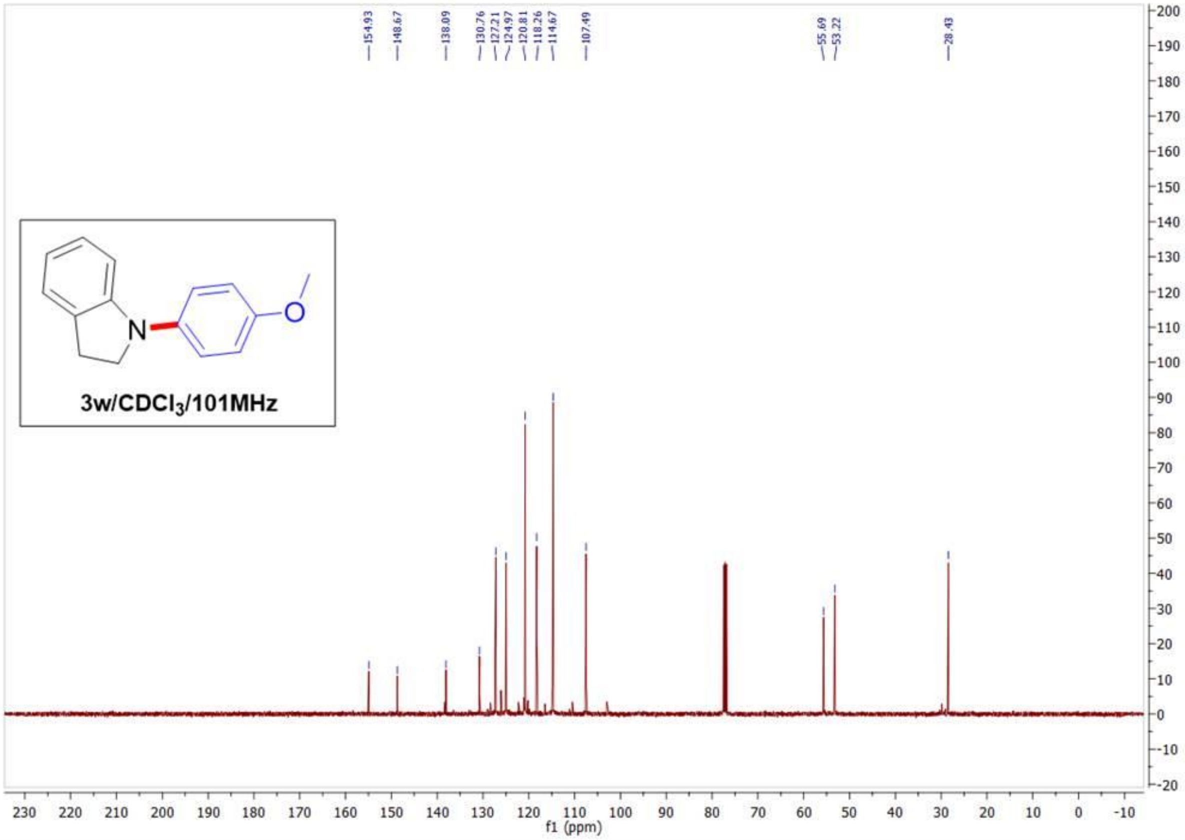
154.93
148.67
138.09
130.76
127.21
124.97
120.81
118.26
114.67
107.49
55.69
53.22
28.43
3w/CDCl3/101MHz
f1 (ppm)
